# A machine learning and directed network optimization approach to uncover *TP53* regulatory patterns

**DOI:** 10.1016/j.isci.2023.108291

**Published:** 2023-10-26

**Authors:** Charalampos P. Triantafyllidis, Alessandro Barberis, Fiona Hartley, Ana Miar Cuervo, Enio Gjerga, Philip Charlton, Linda van Bijsterveldt, Julio Saez Rodriguez, Francesca M. Buffa

**Affiliations:** 1 Department of Oncology, Medical Sciences Division, University of Oxford, Oxford, UK; 2 Department of Epidemiology & Biostatistics, School of Public Health, Imperial College London, London, UK; 3 Nuffield Department of Surgical Sciences, University of Oxford, Oxford, UK; 4 Heidelberg University, Faculty of Medicine, Heidelberg University Hospital, Institute for Computational Biomedicine, Heidelberg, Germany; 5 MRC Laboratory of Molecular Biology, Francis Crick Avenue, Cambridge, UK; 6 Department of Computing Sciences, BIDSA, Bocconi University, Milan, Italy

**Keywords:** Regulatory networks, directed networks, causal inference, mutations, cancer systems biology, machine learning, TP53, trascriptomics, regulon

## Abstract

TP53, the *Guardian of the Genome*, is the most frequently mutated gene in human cancers and the functional characterization of its regulation is fundamental. To address this we employ two strategies: machine learning to predict the mutation status of *TP53from transcriptomic data*, and directed regulatory networks to reconstruct the effect of mutations on the transcipt levels of *TP53* targets. Using data from established databases (Cancer Cell Line Encyclopedia, The Cancer Genome Atlas), machine learning could predict the mutation status, but not resolve different mutations. On the contrary, directed network optimization allowed to infer the *TP53* regulatory profile across: (1) mutations, (2) irradiation in lung cancer, and (3) hypoxia in breast cancer, and we could observe differential regulatory profiles dictated by (1) mutation type, (2) deleterious consequences of the mutation, (3) known hotspots, (4) protein changes, (5) stress condition (irradiation/hypoxia). This is an important first step toward using regulatory networks for the characterization of the functional consequences of mutations, and could be extended to other perturbations, with implications for drug design and precision medicine.

## Highlights

• Machine learning predicts *TP53* mutation status using mRNA expression of target genes (regulon)
• *TP53* regulatory activity inferred for different mutations in CCLE and TCGA using directed causal networks
• Similar regulatory networks are observed when same mutation, deleterious function, and hotspot
• Same stress TP53 perturbation (hypoxia/irradiation) results in similar regulatory activity

## Graphical abstract

**Figure:**
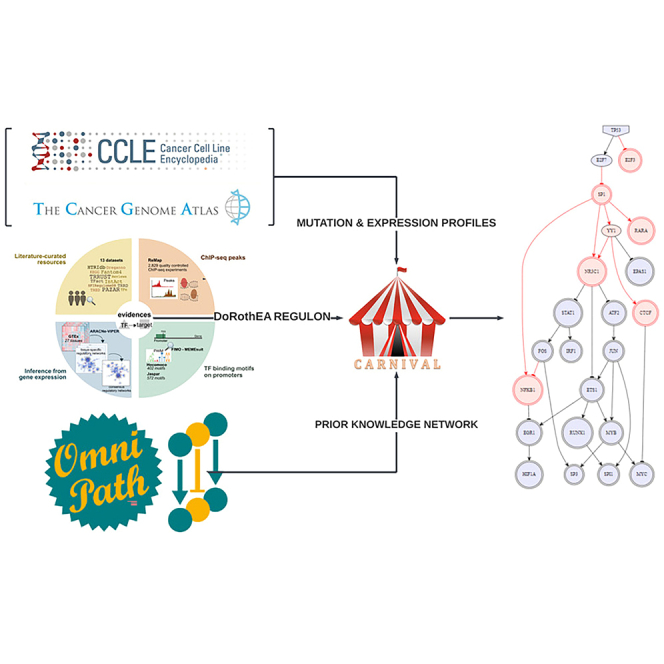

## Teaser

Molecular network; Cancer systems biology; Machine learning

## Introduction

Regulation of gene expression is critical to a diverse array of biological processes in health and disease. Dynamic transcriptional changes drive cell fate decisions in development, disease, and in response to drugs and mitogens. Transcription factors (TFs) are master regulators of gene expression. Sequence-specific TFs can bind to exact regions of the DNA to facilitate transcription initiation of their target genes,^1^ known together as the *regulon*. In cancer, driver mutations have an impact on TFs and many of the key tumor-suppressor genes are TFs or cofactors.^2^ Such mutations lead to structural changes and alter the DNA-binding capacity of the respective TF, impacting their regulon. To prevent targeting cancer agents only to a limited set of molecules or mutations, and to broaden our possibilities for re-purposing of existing drugs, it is critical to understand these networks and to explore the full downstream functional implications of mutations.

Gene networks are a powerful tool to study complex diseases such as cancer^3^^,^^4^^,^^5^^,^^6^^,^^7^^,^^8^; however, they have not been applied systematically to characterize different mutations. Different approaches can be chosen for gene networks analysis and inference, which provide different insights. For example, association networks have been used extensively, while their predictive ability is limited, the associated network metrics (e.g., communities, hubs) can be used to infer gene function (see for example^9^^,^^10^). Directed networks, on the other hand, offer the possibility of representing causality, with inclusion of mode of regulation and differentiation between direct and indirect effects.^11^^,^^12^ Here, we present a causal network approach to analyze the functional consequence of mutations in TFs, and evaluate its utility to identify the impact that different mutations have on the *TP53* regulatory network (regulon).

This wild-type nuclear tumor protein $\left(p53^{WT}\right)$ , also known as the *Guardian of the Genome*, is central to human biology and a tumor suppressor gene. It acts to block cell cycle progression^13^ in the presence of DNA damage to promote repair or in the case of non-repairable DNA damage to enable programmed cell death through controlling a set of genes required for these processes.^14^ *TP53* can induce growth arrest or apoptosis depending on the duration and type of stress, the cell type and other physiological circumstances. The type of functional differentiation, the selection between cell-cycle arrest or apoptosis, based on regulatory networks with a four module model after ionizing radiation has been presented in^15^ which suggests that unique feedback loops in sequence can result in multi-phase behaviors dynamically. Additionally, ^16^ a Boolean model of cell fate decision in DNA damage repair was given between three phenotypes: apoptosis, senescence, and autophagy, and that checkpoints in the p53 pathway can regulate the induction of different phenotypes.

At present, hundreds of mutations have been identified for the *TP53* gene^17^ that lead to structural changes that destabilize p53 structure and alter its DNA-binding capacity and ability to regulate target genes through interaction with transcription regulators and chromatin complexes.^18^ It is important to notice that *TP53* mutation status is not always the only determinant, as p53 function can be modulated via alternative mechanisms which impact its upstream regulators. For example, a gain or loss of function of *MDM2* gene would impact, negatively and positively respectively, on *TP53* stability.^19^ If a mutation renders *TP53* unable to control the expression of its target genes, this can play a critical role in cancer initiation and progression.^20^ While several studies have described p53’s effects on the tumors’ transcriptome and proteome,^21^^,^^22^^,^^23^^,^^24^^,^^25^^,^^26^^,^^27^^,^^28^^,^^29^^,^^30^^,^^31^^,^^32^ the implications for the different types of *TP53* mutations on these, are still understudied. Thus, it is timely to carry out a systematic analysis of genomics datasets to explore different methodological approaches to study p53 function in human tumors, and the effect of mutations. Importantly, due to the heterogeneous nature of *TP53* mutations, the ability to distinguish deleterious (i.e., a mutation that renders the protein non-functional) from other *passenger* mutations is of great importance. The differences and functional implications between these mutations could be one of the key reasons why in most cancers *TP53* mutation status have not been applied in the clinic to predict patients response to therapy and to guide clinical practices. Indeed, to be able to link certain types of p53 mutations (missense, deletion, hotspot etc) with their function is paramount to precision medicine. It provides the first building block to translational data science in the heterogeneity of somatic mutations in cancer, being able to map the transcriptome of a patient to regulatory networks. Thus, we can then understand how different and acquired clonal evolution from a mutational perspective can be utilized to provide better therapeutics by exploiting individual-specific up/down regulated pathways with precision.

**Figure 1:**
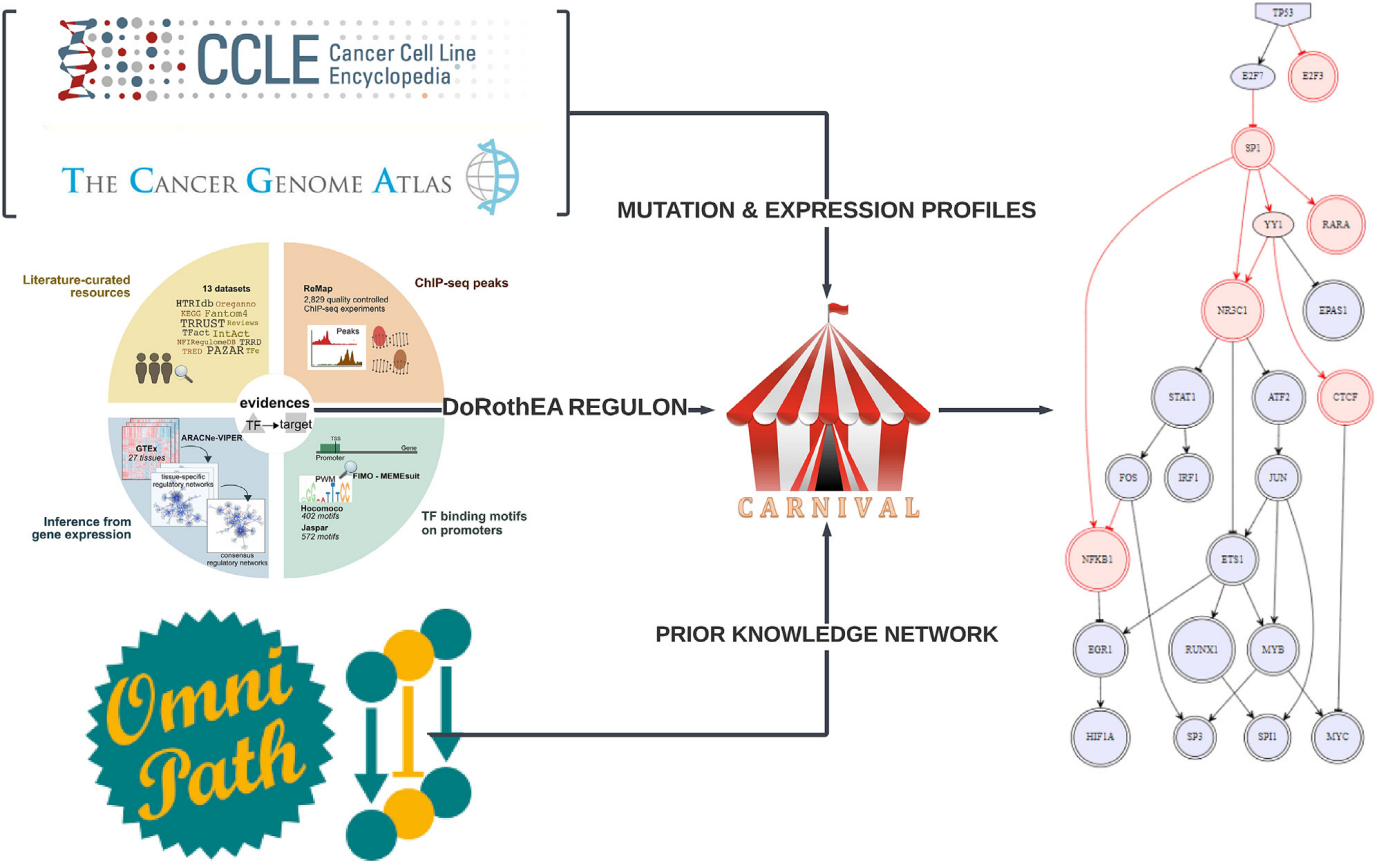
Visual summary of the directed gene network approach First, expression and mutation profiles for the transcription factor (in this case *TP53*) are collected via established databases for cell-lines (CCLE) and tumor samples (TCGA). The regulon, as a set of target genes, is then extracted from DoRothEa, emanating from different sources of databases from experiments in cancer, with different levels of confidence (A–E). In addition, the prior knowledge network (PKN) as a collection of interactions is extracted from OmniPath. These three components are then used as an input in the CARNIVAL pipeline, where an optimization model reconstructs the PKN based on the perturbation and the given expression profile. In this way, we optimize one network per mutation across each sample, and are able to compare them for topological features based on the annotation each time.

Using both machine learning (ML) and directed graph network representation methods, and exploiting the abundance of omics data now available with both whole-transcriptome information and recorded mutation status, we set to study in a systematic manner the impact of different *TP53* mutations on the expression of its regulon. To ensure that our reconstructed networks reflect the downstream effect of the real spectra of *TP53* mutations occurring in cancer, and investigate how these impact *TP53* function and clinical outcome, we interrogated 1,457 cell lines across 22 cancer types from the Cancer Cell Line Encyclopedia (CCLE) and 12,531 cancer samples across 54 cancer types and sub-types in The Cancer Genome Atlas (TCGA) databases. First, we assembled a comprehensive list of validated and predicted targets, ranked by level of evidence, using DoRothEA,^33^^,^^34^ specifically inferred for cancer. We then followed two approaches: (1) using state-of-art ML methods to ask if we could build a predictor of *TP53* mutation, and mutation types, based on changes in the transcription data, and (2) we asked if a directed gene networks approach, enabling to infer causality, to reconstruct *TP53* regulatory networks (Figure 1) could distinguish between different *TP53* mutations, or *TP53* differential activation in different conditions, such as hypoxia and irradiation. In the second approach, the data were stratified based on perturbation: the TF (in this case *TP53*) is perturbed based on the deleterious (or not) function of the mutation for the gene (correspondingly hypoxia versus normoxia and irradiation or not, for the RNA-seq experiments), and the gene expression profile with the topology of the network is reconstructed using mathematical modeling and optimization, and then analysed to extract gene signature of specific mutations (Figure S1). This single-sample approach of network optimization enables the study of single mutations appearing in either cell-lines or tumor samples. Although more general validation would be required, this methodological framework could allow *in silico* monitoring of the functional impact of specific mutational events in single tumors at diagnosis, or *de novo* mutations occurring during treatment, thus opening new therapeutic options for cancers that are resistant to current therapeutic regimes. Additionally, the framework can provide insights at a population level, by better understanding the sensitivity of TF regulation stratified by cancer types across thousands of samples, and could be applied to diseases besides cancer.

## Results

### Expression of *TP53* is heterogeneous and non-predictive of mutation status

The *TP53* mutational landscape across human cancers and cell lines is highly heterogeneous (see^35^^,^^36^^,^^37^^,^^38^). While carcinogenesis implies biallelic loss of functional tumor suppressor genes, the most typical *TP53* mutation configuration is a single *TP53* mutation with loss of the remaining *TP53* allele through a large-scale deletion on chromosome 17p.^39^^,^^40^ Additionally, mutant p53 can have a dominant negative effect over $p53^{WT}$ and/or gain of function activity independently of the wild-type protein.^35^ There is also evidence that single mutations of *TP53* are associated with loss of a single allele (∼ 2/3 of tumors) and a high distortion CNV, whereas tumors with more than two mutations usually retain both alleles (diploid in almost one-third of cases).^21^

In our analysis we found that *TP53* was mutated in 4,250/12,531 (34%) of the TCGA cancer samples and 898/1,457 (61%) of cell lines, with some cancer types showing strong mutational frequency (such as ovarian, lung, and glioblastoma) and others much less so (Figures S2A and S2B). Approximately 65% of all found mutations were missense mutations (point mutations where a single nucleotide change codes for a different amino acid) in both TCGA and CCLE (Figures S3A and S3B). This is in agreement with previous reports.^21^^,^^41^^,^^42^^,^^43^ Mutation type and deleterious function of mutation respectively were extracted from CCLE and TCGA, and we considered known hotspot mutations with protein changes as follows: p.R175H, p.R248Q, p.R273H, p.R248W, p.R273C, p.R282W, p.G245S as those characterized in^44^ as missense mutations. Finally, both analyses of TCGA tumor samples and CCLE cell line data show that only 10% of samples contain more than a single mutation for *TP53*.

First, we asked the simple question of how these different mutations correlated with gene expression of *TP53*, measured as mRNA levels. We found that expression varied across different mutations, with some mutations resulting in higher expression with respect to WT status, and others lower (Figures S4A and S4B). Interestingly we observed a very high concordance between cell lines and cancer samples. Samples with frameshift mutations (deletions/insertions) and nonsense mutations showed generally lower expression than WT samples in both CCLE and TCGA. In-frame (deletions/insertions) and missense mutations showed higher levels of expression, while other mutations showed levels of expression similar to WT; post-hoc tests that report the differences between each group for databases can be found in the supplementary information. Samples with hotspot mutations and deleterious mutations showed respectively a significantly higher (Figures S5A and S6A) and lower (Figures S5B and S6B) expression of *TP53* than other mutations. We also looked at Copy Number Variation (CNV), Figure S7. In CCLE, copy number gain and diploidy were associated with higher median expression across WT and mutated samples. In TCGA, the highest expression in mutated tumor samples was found in amplification, whereas besides deep and shallow deletions in wild-type samples, all other types of copy number variation had similar but higher expression values (Figure S7).

### Association with prognosis supports functional heterogeneity of *TP53* mutations

*TP53* mutations have been associated with cancer progression and poor prognosis^45^; however, the landscape is complex (for a review see e.g.,^46^). In some cancers, such as hematological malignancies or ovarian cancer, *TP53* status is also used to guide the treatment strategy. In others, such as breast cancer, some evidence of association of *TP53* mutation with poor survival have been produced, however the landscape is not entirely clear,^38^ and it is largely dependent on treatment and accurate characterization of mutation type. Some specific mutations can have a significantly different impact on survival than others; for example, missense mutations within exons 5 to 8, corresponding to the DNA binding domain, have been shown to correlate with poorer survival than silent mutations in this region or no mutations.^47^

Building on this evidence, we asked whether stratifying patients by any of the different types of *TP53* mutation would allow us to better correlate *TP53* with patient disease outcome than simply grouping patients by wild-type or mutant status. We interrogated TCGA datasets and considered the following sample settings (Figure S8): Figure S8A samples with *TP53* mutant versus WT status, Figure S8B samples carrying missense mutations vs. other mutations (excluding WT samples), Figure S8C samples carrying missense mutations vs. other mutations vs. WT (3 groups), Figure S8D samples with deleterious mutations vs. non-deleterious mutations and WT (3 groups), Figure S8E samples carrying hotspot mutations vs. non-hotspot mutations and WT (3 groups), Figure S8F samples with missense deleterious mutations vs. missense non-deleterious, Figure S8G hotspot deleterious vs. non-hotspot deleterious, Figure S8H hotspot vs. non-hotspot and WT (3 groups) and finally Figure S8I hotspot vs. non-hotspot (no WT).

The results show that *TP53* mutation was associated with worse prognosis (p<0.0001, Figure S8A). Furthermore, samples carrying missense mutations were associated with worse prognosis when compared with samples with a non-missense mutations and WT (p<0.0001, Figure S8C). Deleterious mutations showed worse prognosis to non-deleterious mutations and WT groups (p<0.0001, Figure S8D). Samples with hotspot mutations had worse prognosis than samples with non-hotspot mutations and WT groups (p<0.0001, Figure S8E). Furthermore, specifically for missense mutations when comparing the deleterious versus the non-deleterious, we also observe worse prognosis for the non-deleterious (p=0.00013, Figure S8F). This is in agreement with previous research^21^ as a missense mutation, changes the structure of the p53 protein but also makes the protein negative dominant on the WT version (which is a tumor suppressor). Finally, when comparing hotspot and non-hotspot but both deleterious, the worse prognosis is seen in the case of non-hotspot deleterious cases, although the numbers are small (p=0.016, Figure S8G). Furthermore, we compared the survival curves in two additional settings in relation to *TP53* hotspot mutations (as those appear in^44^). We can see (Figure S8H) that when we include the WT samples, we clearly see a difference in survival (p << 0.05) but as shown in Figure S8I, by removing the WT samples from the data this difference is no longer significant. Finally, we can see that when comparing missense mutations alongside WT samples, deleterious mutations and hotspot mutations for *TP53* (Figure S9), WT samples have as expected better survival than missense mutations, followed by deleterious and hotspot mutations. These results are complex, but taken together confirm that *TP53* mutational status correlates with clinical outcome and importantly, that specific types of mutations affect patient survival differently.

### Using machine learning, we are able predict *TP53* mutation status (WT/MT) based on the expression of *TP53* regulon, but not the type of mutation

An initial principal component analysis (PCA) of the mRNA expression of the genes in the regulon of *TP53* (Figure S10) showed that the first principal component accounts for 15.6% of the variation while the second for 11.5%. This confirms differential regulation of gene expression of *TP53* target genes, but this global variation was not associated with mutation. Hierarchical clustering analysis confirmed these results (Figure S11) showing different patterns of up or down-regulation for different groups of regulon genes, but not an immediately clear correlation with mutation status. These results together indicate that although the expression of *TP53* itself is not predictive of mutation status, specific gene expression features in the clinical samples could be.

To support this, a published basic signature of four genes (*CDC20, CENPA, KIF2C,* and *PLK1*)^21^ has been shown to be significantly correlated with the presence of *TP53* mutation in clinical samples. However, it is not easy to draw conclusions as these genes were not validated targets of *TP53*. On the other hand, these four genes were characterized by higher expression in *TP53* mutated samples with respect to *TP53* WT, suggesting that *TP53* represses their expression when active. Many of the repressed genes do not contain binding sites, hence they tend to be not represented in regulon databases, and this could explain why they have not been validated as target genes in previous studies. Thus, we asked first, using an Elastic Net-based approach, if we could develop a predictor for these four genes, and then evaluate its performance with respect to a predictor based on *TP53* deposited targets from the DoRothEA database. Finally, we asked if these gene signatures could predict not only mutation status but also the type of mutation present in the specific sample/group of samples, using both Elastic Net and an XGBoost (Gradient Boosting) classifier.

**Figure 2:**
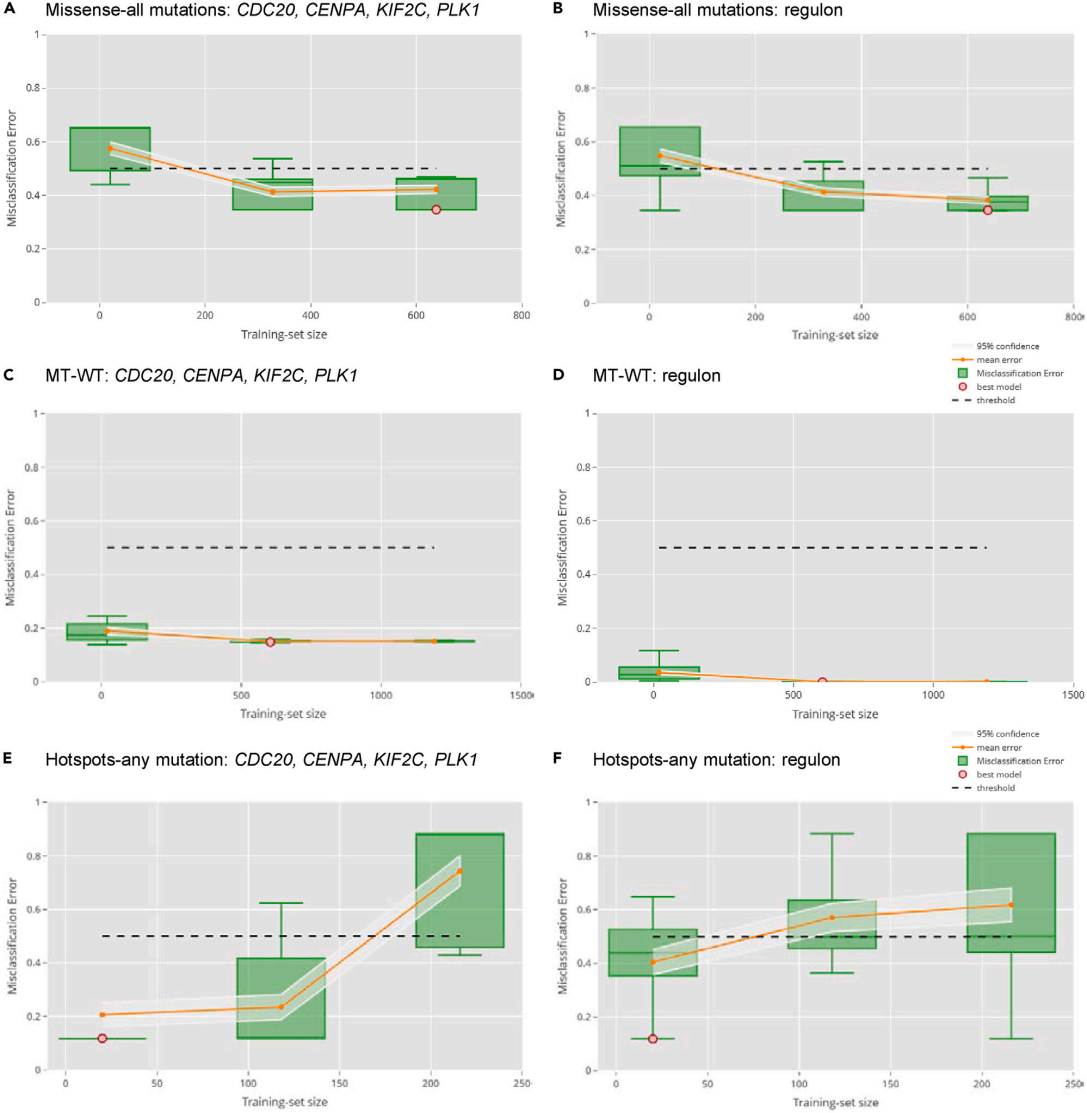
Performance of models predicting *TP53* mutation in cell lines (CCLE) using RNAseq The models were built using a minimal four-gene signature and a comprehensive regulon from DoRothEA as described in the text. (A–F) Penalized regression was used with multiple settings. An Elastic Net model (see STAR methods) is built in cross-validation for different train-test combinations and misclassification error assessed. The models were trained to predict three different p53 mutational features: (A and B) Missense vs. any mutation, (C and D), WT (wild-type) vs. MT (mutated) and (E and F) hotspot p53 mutations. In each plot, the x-axis represents the different training set sizes while the y-axis shows the accuracy measure (i.e., the misclassification error) used to assess the performance of the fitted models. The mean error and the associated confidence interval are also reported for each training set size. Each green dot in the plots corresponds to a trained model. The red dot represents the best model selected in cross-validation (see STAR methods). Different training set sizes are used, and the one providing prediction error with the lowest upper confidence interval was chosen. The best model is then selected so as to have the minimum misclassification error.

**Figure 3:**
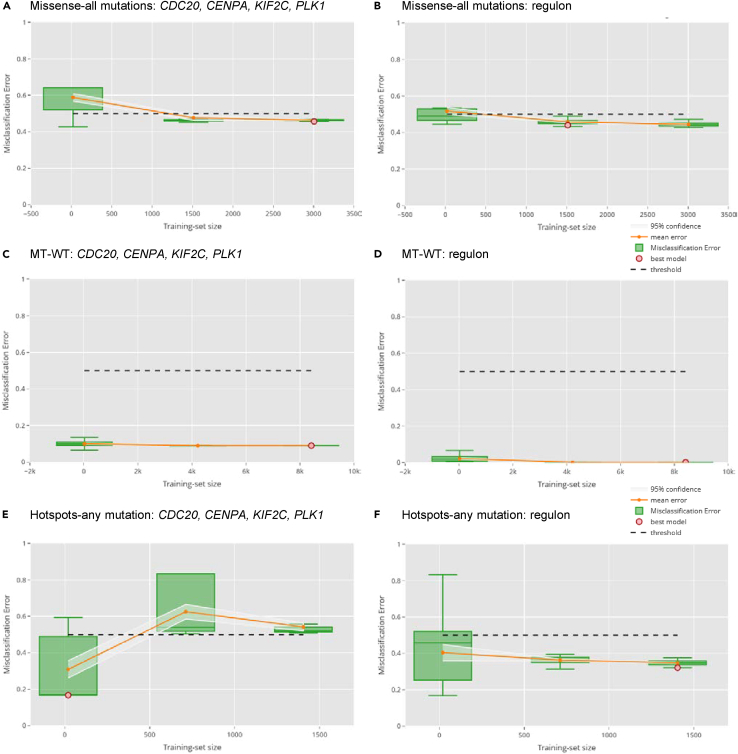
Performance of models predicting *TP53* mutation in cancer samples (TCGA) using RNAseq The models were built using a four-gene signature and the regulon of *TP53* from DoRothEA as described in the text and the methods (See STAR methods): (1) (A and B) Missense versus all other types of mutations (no WT samples included), (2) (C and D) WT versus any mutation, and (3) (E and F) hotspot p53 mutations versus all other non-hotspot mutations (no WT samples included). In each plot, the x axis represents the different training set sizes while the y axis shows the accuracy measure (i.e., the misclassification error) used to assess the performance of the fitted models. The mean error and the associated confidence interval are also reported for each training set size. Each green dot in the plots corresponds to a trained model. The red dot represents the best model selected in cross-validation (see STAR methods). Different training set sizes are used, and the one providing prediction error with the lowest upper confidence interval was chosen. The best model is then selected so as to have the minimum misclassification error.

First, we considered the four genes previously identified and the *TP53* regulon, with the following aims: (1) prediction of missense mutation with respect to other mutations, (2) prediction of any *TP53* mutation, and (3) prediction of hotspot mutations. We did this in a cross-validation setting and varied the train-test set proportions during the re-sampling to ensure that the size of the training dataset did not affect significantly the results (Figures 2A–2D and 3A–3F, and see STAR methods). We observed that both the previously identified set of four genes and a comprehensive regulon signature could predict well the presence of *TP53* mutation of any type, in both the cell line data (Figures 2C and 2D) and cancer samples (Figures 3C and 3D). This confirms, as expected, that the regulon usage is indeed generally different in *TP53* mutant tumors with respect to *TP53* WT. Specifically, the four-gene model achieved misclassification of 15%, and a model built with the extended set of regulon genes achieves 0% mis-classification, suggesting that the use of the full regulon provides an advantage with respect to using only the four-gene signature. When we tried to predict missense mutations against any other mutation, the model performance deteriorated (Figures 2A, 2B, 3A, and 3B). The average misclassification was between 35% and 40% for CCLE data and above 40% for TCGA data. Eventually, using this approach we could not build a robust predictive classifier of the type of *TP53* mutation. Importantly, differences might be there between the types of mutations, but not strong or stable enough to allow a robust predictor to be built. Additionally, we attempted to calculate a binomial classifier between the hotspot mutations of *TP53* (as those appear in^44^) and any other mutation in both CCLE and TCGA. In this case the signal was very good and we could detect differences in the regulon usage. Both of the signatures performed well in CCLE cell lines 2(e),2(f), averaging around 10% miss-classification error, whilst in the TCGA case the four-gene signature performed better than the regulon (less than 20% miss-classification error as opposed to approximately 30% for the regulon) Figures 3E and 3F.

To further investigate whether a structural problem occurred that could explain the fact that a linear classifier could not predict mutation types, we probed a non-linear classifier (XGBoost, a tree-based approach that uses a regularizing gradient boosting framework) in both CCLE and TCGA. Using a *softmax* objective function we were, again, unable to construct a classifier that can predict with high accuracy the *TP53* mutation type based on the mRNA expression of its DoRothEA validated regulon (targets). The misclassification error in the test set was in both cases similar (approximately 38%, see supplementary information).

### *TP53* mutations of the same type or with same deleterious function show similar target regulatory network in cell lines and tumor samples

To investigate the different functional implications of different types of *TP53* mutations at a global scale, we asked if we could observe global network rewiring in *TP53* mutant cases, and in different mutation types. To address this we used network modeling with a directed graph as a base. First, we assembled a prior knowledge network (PKN) for *TP53* regulon using OmniPath^48^ for the human genome. We considered only targets with the known mode of regulation, and where the source to target information was available (14,855 interactions and 586 target genes). We then processed the expression and mutation profiles from CCLE and TCGA. Since we are interested in the downstream effect of *TP53*, we used the CARNIVAL pipeline to map the expression data as activation/inactivation projected onto the PKN and reconstruct it using optimization modeling (a Mixed-Integer Linear Problem).

In first instance, we defined the perturbation of *TP53* based on its deleterious status as a knock-down when true, and as active when false (based on CCLE and TCGA Deleterious feature annotation for each single mutation). CARNIVAL requires three sources of information to be initialized: (1) the sign of the applied perturbation (active or not) which in our case is the *TP53* mutation, (2) PKN, and (3) the expression matrix of the regulon of the TF. All these three inputs were acquired by using (1) the deleterious (o not) annotation of the mutation, (2) the PKN using OmniPath and DoRothEA, and (3) the mRNA expression from the relevant databases (CCLE, TCGA and others). The expression matrix is converted to Normalized Enrichment Scores (NES) per sample (see STAR methods). Using CARNIVAL we then reconstruct the topology and gene activity profile for the regulon. The network is optimized by minimizing the mismatch between the predicted state of each gene according to the consistency rules imposed by the optimization model’s constraints and the NES scores. This reconstruction changes effectively the topology of the network, i.e. the mode of regulation between the nodes, as well as the genes that are involved.

**Figure 4:**
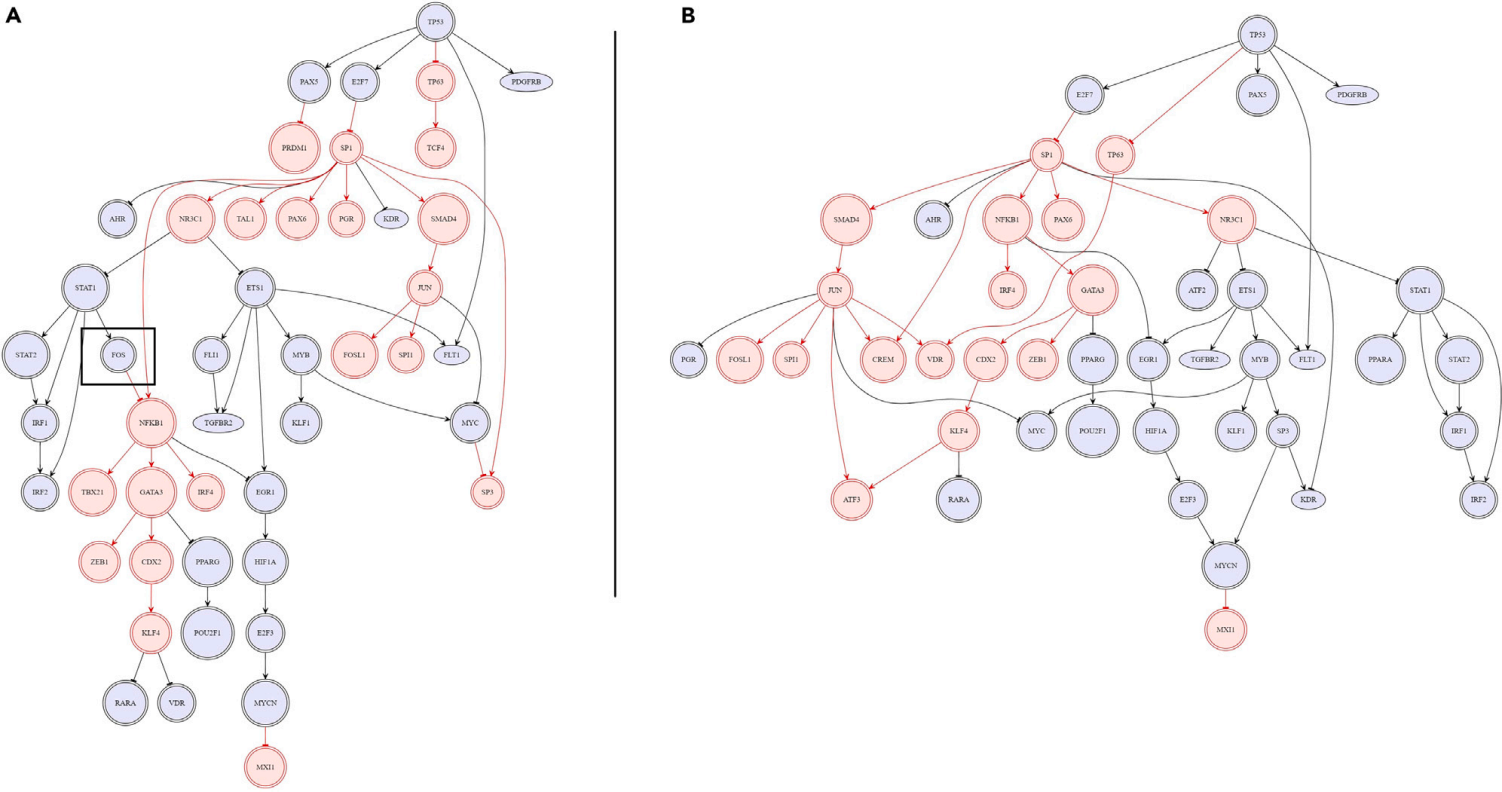
Illustration of our network comparison technique using two reconstructed networks in two distinct samples from breast cancer (A) An optimized based on expression and mutation information breast cancer cell line sample carrying a missense p53 mutation (protein change: p.E224K, cell line: CAL148, deleterious: False) and (B) equivalently a missense p53 mutation sample from a breast cancer cell line (protein change: p.E285K, cell line: BT474, deleterious: False). On top we see the perturbation node, our transcription factor *TP53* and its downstream DoRothEA target genes. We seek to understand what topological differences (both activation/inactivation and mode of regulation) exist between these two networks to calculate a percentage of similarity based on the edge intersection of these two networks, treating them as graphs. For instance, on the left network (A) and denoted with a framed rectangle, an activating arrow from *STAT1* to *FOS* exists whereas this edge is missing completely from network (B). These kinds of differences are taken into account to compute the similarity score (see STAR methods). The fraction of common edges found in both networks over the maximum number of edges (in the largest network of the two) gives the percentage of similarity. These common edges include the same starting node, end node and mode of regulation.

**Figure 5:**
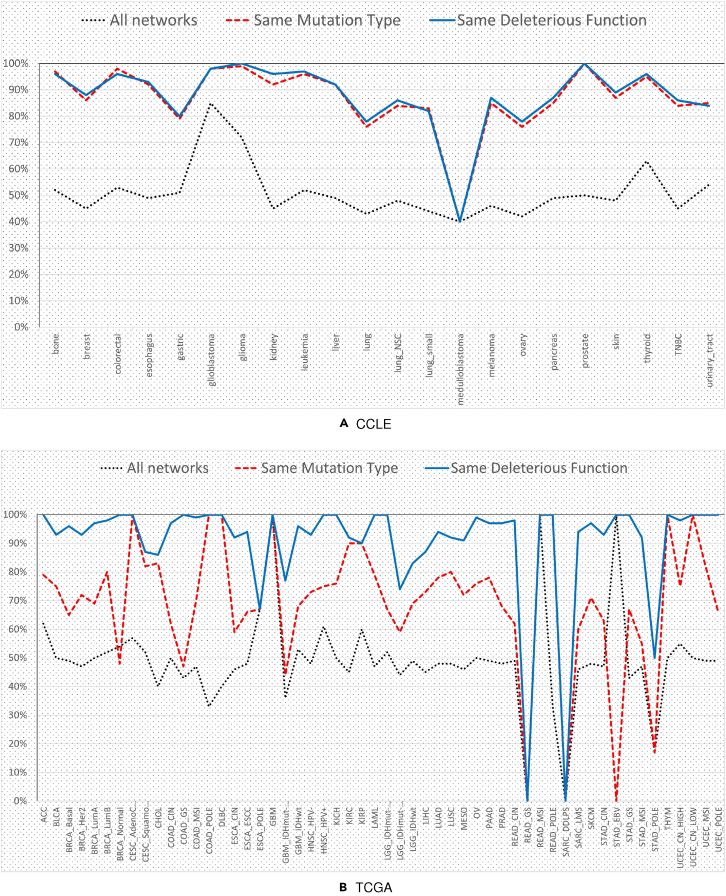
Similarity of the directed networks reconstructed for different *TP53* mutations in CCLE and TCGA samples (A and B) The different cancer types (x axis) in CCLE (A) (for a cut-off of at least 50% similarity) and TCGA (B) and the percentage of networks (y axis) that are similar across this cut-off, across three different settings: (1) all networks compared, (2) same mutation type, and (3) same deleterious function of mutation for *TP53*. It is evident that the similarity of the networks improves drastically across last two settings as opposed to the general first setting that does not take into account any feature when comparing the networks. These two plots together summarize the conclusion that when taking into account p53 mutation type or deleterious function of mutation, the regulatory profile of the transcription factor *TP53* is significantly more similar than by grouping randomly, in cell lines and tumor samples. Of note, in (B), sub-types STAD_EBV, SARC_DDLPS and READ_GS have no same *TP53* mutation type pair identified in the data, thus the percentage is 0%. Additionally, SARC_DDLPS and READ_GS also do not contain a pair of same deleterious *TP53* function. Finally, for CESC_AdenoCarcinoma,COAD_POLE,DLBC,GBM,READ_MSI,READ_POLE, at least 50% of the compared network pairs had 100% similarity (identical graphs). The full data is shown in Tables S1 and S2 and radar plots Figures S13–S20.

We then compared the networks calculating a similarity score (see STAR methods) between all pairs of optimized networks generated in both CCLE and TCGA databases Tables S1 and S2. An example is given in Figure 4 where we compare two different networks representing the downstream effect of different perturbations on *TP53*. According to our analyses, the proportion of the similar networks in both CCLE and TCGA significantly improves when we move from general/global similarity (all possible combinations of networks compared without filtering for a specific p53 feature) to either comparing networks reconstructed in samples with the same type of mutation or deleterious functional impact of the mutation for *TP53*. This is true for all cut-off levels of similarity considered (25, 50, 75, and 90%). It is evident from Figure 5 (this figure was generated using R package UpSetR^49^) that the similarity between networks is lower when comparing all networks than the when comparing samples with the same type of mutation and same deleterious function, for a cut-off of 50% similarity score. For hotspot mutations of p53, the results show less association between the feature and the downstream graph similarity on the optimized network. This might be an effect of fewer observed cell lines and tumor samples harboring one of the analyzed hotspot mutations. In the case of TCGA, 36/54 sub-types have a greater percentage of networks reaching the 75% cutoff similarity threshold when only cases with the same type of mutation are included than in the general case. This ratio improves when we compare against same deleterious feature of mutation to 45/54. Equivalently, in CCLE, 19/22 cancer types improve the total number of similar networks when we set the cutoff similarity threshold to 75% and above and comparing the general case with same type of mutation. This also improves when we compare against the same deleterious feature of mutation in 21/22 cancer types. Notably, in CCLE, Leukemia projects the biggest similarity gain moving from the general case to the same type of mutation (up by 33%). In Figure S12 we see plots for entirely different comparison scenarios. The plots show that different mutation type and different deleterious function of the mutation generate different networks as opposed to the general case where we compare all networks as baseline, and that when the pair of compared networks comes from a mutation of the TF which corresponds to the same protein change, we also get very similar network topology. This further amplifies our initial statement that grouping by same mutation type or deleterious function of mutation for *TP53* shows highly more similar downstream regulation, than by random grouping.

### Application of the methodological framework under irradiation and hypoxia

In order for us to investigate the inferential ability of our framework in a perturbing event other than somatic mutations, we applied it on different cell line conditions using our RNA-seq experiments in irradiation and hypoxia. Given that we know already that irradiation and hypoxic stress activate *TP53*, we use this information to probe whether our framework can capture strongly differential signal on its regulon (downstream regulated gene targets). Indeed, we show that the TF changes its mode of regulation between the target genes based on the stress condition each time and that inverse stress conditions (e.g., normoxia vs. hypoxia) show drastically different regulation of the target genes.

#### Network modeling on WT *TP53* lung cancer cell lines under radiation reveals different downstream effects on its regulon based on p53 perturbation as an effect of irradiation

We investigated the downstream effect of different perturbations of p53 using a WT *TP53* lung cancer cell line (H460) between no treatment (0 h) and after irradiation. The experiment is a time course (0 = pre-radiation, and 2, 6, 12, 24 h post 2 Gy radiation) with 3 cell lines (H460 Parental, and 2 radio-resistant - H460_50B and H460_60A). The results indicate that after irradiation (>0 h), when p53 is activated, the signal downstream changes drastically in comparison to samples before irradiation (where p53 is inactive). The networks compared at random (all network pairs) show that the percentage of networks that meet the 25, 50, 75, and 90% cut-off similarity scores is 100%, 67%, 67%, 36% correspondingly. When we compare only irradiated with non-irradiated cell-lines, the network similarity is 100%, 0%, 0%, 0% for all four cutoffs equivalently (Figure S21). Therefore, this is a strong indication that irradiation changes drastically the regulatory profile for *TP53* in the analyzed lung cancer cell-lines.

#### Breast cancer cell line experiments reveal the differential downstream regulatory profile of *TP53* based on hypoxic conditions

Next, we investigated the effect of hypoxia on *TP53* using RNA-seq in two independent experiments. In the first experiment, a panel of four breast cancer cell lines (MCF7; MDA-MB-231; HCC1806; MDA-MB-468) were exposed to 1% or 0.1% $O_{2}$ and collected at 24 h and 48 h time-points. The second, smaller experiment (45 samples) focused on only two of these cell lines (MCF7 and MDA-MB-231), and was limited to a single hypoxia condition (24 h, 1% $O_{2}$). In the first and larger experiment (360 samples), we found that the percentage of networks with similarity score of 25, 50, 75 and 90% was 100%, 55%, 53% and 30% correspondingly. When we filtered for inverse conditions (hypoxia/normoxia) we saw the similarity reduced dramatically to 100%, 0%, 0%, 0% equivalently for each cut-off percentage.

When we compared the networks from the smaller experiment (45 samples/networks) we found that the percentage of networks with similarity greater or equal than 25, 50, 75, 90% was found to be 100%, 49%, 36%, and 25% respectively (in the general random comparison pooling case). Filtering the comparisons only between samples that correspond to inverse oxygen conditions, that is, either 0.1 or 1% hypoxia on one side and normoxia on the other, we found that the percentage of networks with similarity greater or equal than 25, 50, 75, 90% fell drastically to 100%, 0%, 0%, and 0% respectively (Figure S22).

### Post-optimization network analysis reveals gene sets characteristic of each mutation type in CCLE and TCGA

**Figure 6:**
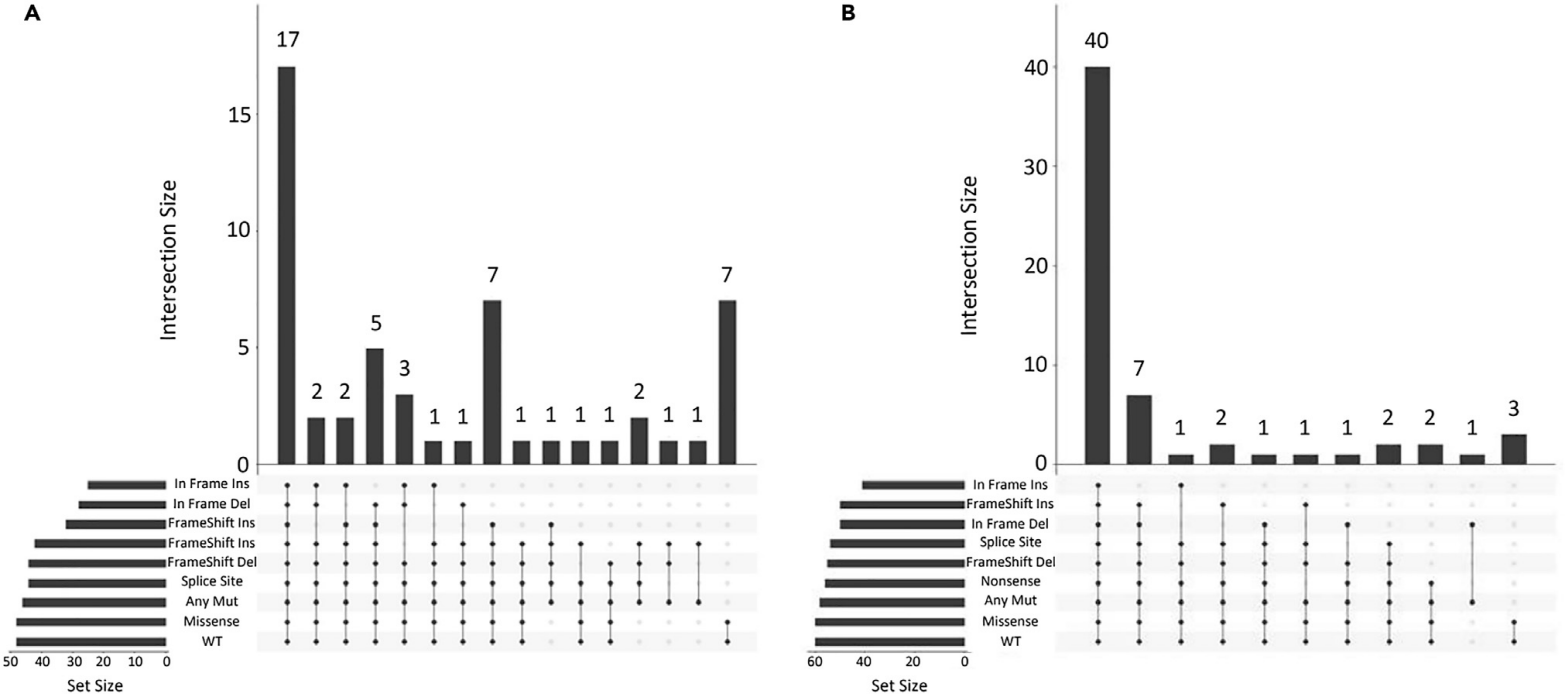
Common genes across signatures extracted from the directed networks reconstructed for the different *TP53* mutations (A and B) *TP53* mutational meta-signatures (across all cancer types) for TCGA 5(A) and CCLE 5(B) derived using Louvain community detection (see text). Plots are done using *R* package and stratified by mutation. The signatures fluctuate in the number of genes involved approximately from 40 to 60 genes per mutation, in both cell lines and tumor samples. We can see the similarity in the number of genes shared across signatures in both CCLE and TCGA in the first column (all signatures), having 17 common genes in CCLE and 40 common in TCGA. Notably, missense mutations (the most prevalent across cancers) share seven genes in CCLE with non-deleterious signature and three genes with non-deleterious signature in TCGA, highlighting the specificity of the missense signature pan-cancer.

We performed community detection using the well established *Louvain* method^50^ on all optimized networks in both CCLE and TCGA. This method attempts to create a graph partition so that the modularity metric is maximized. A bigger value in the modularity metric means that the identified communities are more tightly connected as independent hubs. We then disconnected the networks across all detected communities and performed maximum betweenness centrality^51^^,^^52^ scoring per community. By the union of all the highest scoring genes per community in terms of the centrality score, we then extracted gene sets for each different mutation type (Figure S1). We then merged the sets per mutation type across all cancer types/sub-types. Hence, we constructed nine *meta*-signatures (pan-cancer), one for each different mutation type for both CCLE and TCGA. We present the distinct intersections of the sets of signatures across all different mutation types in both TCGA (Figure 6A) and CCLE (Figure 6B). Many of these genes are known to be cancer drivers, prognostic factors in cancer or genes linked with response to stress, coherently with *TP53* mutations differential functional role in cancer. We can see that the missense mutation signature, the most prevalent in terms of frequency of occurrence across different cancer types for *TP53*, shares only one gene with deleterious mutations in TCGA and six with non-deleterious in CCLE. This post-network analysis serves as a *visual* mapping of the optimized networks to a list of genes, highly representative of all networks, pan-cancer, per mutation type. In this way, we can see that the networks tend to be different in key-gene composition, something that is not directly observed by just optimizing the respective pool of networks in whole.

## Discussion

We presented a directed network approach to assess the functional effects of the mutational/stress stimuli landscape of the most frequently mutated gene in human cancers, the guardian of the genome *TP53*, across cell lines and tumor samples as well as our in-house RNA-seq experiments in hypoxia and irradiation. To our knowledge, this approach is new since we attempt to evaluate whether different mutations impact the regulon and interacting pathways of a TF in a substantially different regulatory profile, as opposed to simply differentiating expression profiles between WT and MT. Conversely, we are also able to assess whether the same types of mutation cluster across the similarity of the reconstructed networks. Importantly, our approach is not limited to the downstream regulon, but it can also account for potential upstream network rewiring, which can involve other TFs and interacting pathways, and can be also applied to other diseases besides cancer.

To evaluate our approach we used genomics and transcriptomics data from large public databases, together with previous knowledge of network biology. First, we evaluated the efficacy of the gene signature from DoRothEA (the *TP53* regulon) on its ability to predict the status of these differential features in both CCLE and TCGA versus another well-known *TP53* signature. To do this we used state-of-art machine learning (ML), including Elastic Net and XGBoost. Although the regulon Elastic Net classifier distinguished better any type of mutation of *TP53* versus the 4-gene classifier, both were unable to predict effectively the type of the most prevalent *TP53* mutation (missense). Even in the case of training a non linear classifier (XGBoost), we were not able to separate the labeling space with sufficient accuracy. This indicates that ML is not necessarily suffering from a structural approach, but pieces of the puzzle to recover differential regulation are to be found elsewhere.

Thus, we investigated a directed network approach to extract the optimized networks (the regulon of *TP53*) by using perturbation experiments sequentially; each experiment maps to a unique *TP53* mutation and the perturbation depends on the deleterious effect of the variant classification (deleterious: knockdown, non-deleterious: activated). In this way, we were able to infer the topological similarities of all possible pairs of networks, comparing across all *TP53* mutations found in both databases. In turn, we classified the similarity strength based on either random pooling (no underlying common mutational feature/total-general similarity in tables and figures) or against same type of mutation, same deleterious function or an identified *hotspot* mutations for *TP53*.

Indeed, by linking the deleterious function of mutation for the perturbed TF, and by accounting how expression re-wires mode of regulation between the target genes of the TF, we were able to recover a strong signal differentiation under different settings in the regulation. Across different types of cancers, the strength of the signal we infer about the relationship of the mutational status of *TP53* and the status of its regulon downstream was found to fluctuate, with some cancers showing a stronger signal versus other types. However, the overall conclusion is that when we compare the optimized directed networks based on same mutation type or deleterious function of the mutation, or same protein change (Figure S12) and identified hotspot mutations, we obtain more similar networks/similar downstream regulation, as opposed to random comparison across all networks (all mutated samples of *TP53*). This is consistent across 22 types of cancer in cell lines (CCLE) and 54 sub-types of cancer in tumor samples (TCGA), further implying that our methodological approach can unveil the true phenotypic impact and functional characterization of TF regulation *in silico*.

Using established community detection methods and centrality metrics, we were able to extract gene sets/signatures for all optimized networks. By stratifying and unifying per mutation type first, and then per cancer type, we were able to construct meta-signatures for each mutation type, representing the union of all signatures extracted per mutation type across either cell lines (CCLE) or tumor samples (TCGA) respectively. The comparison of the signatures clearly demonstrates that although a significant overlap is observed across the signatures, distinct genes, of which many important oncogenes, for each mutation type differentiate and set the signatures apart. In essence, the gene signatures provide an indirect way to *visualize* almost twenty thousand of optimized networks based on the key gene *players*, as those identified by centrality scores and on a reconstructed topology based on previous knowledge, mRNA expression and mutational profiling.

**Figure 7:**
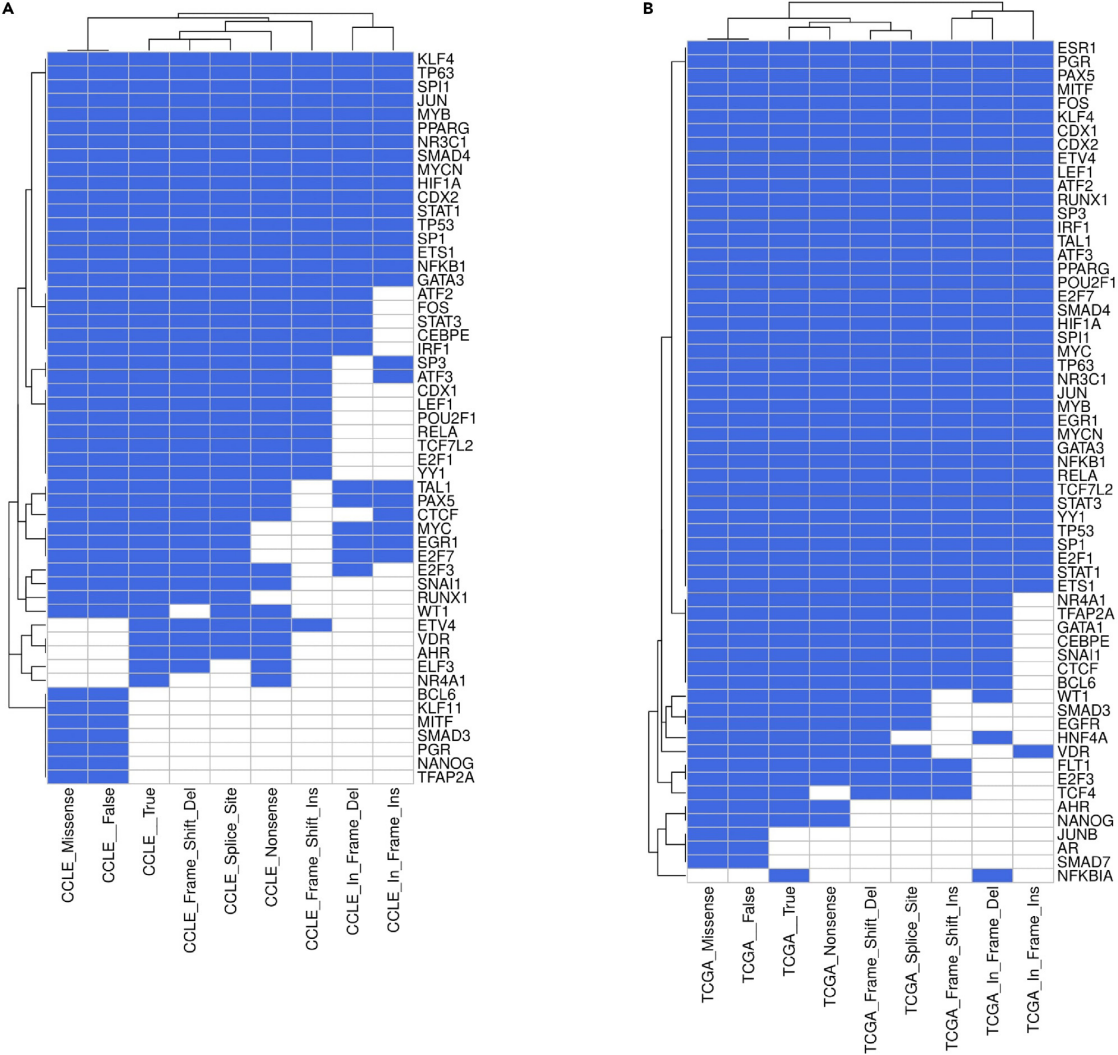
Changes in the regulatory network signature of *TP53* under different mutational backgrounds (A and B) Heatmaps showing predicted changes in CCLE (A) and TCGA (B). Empty cells indicate a predicted loss of interaction between the gene and p53.

Different mutation characteristics of *TP53* appear to drive differences in the regulon (see Figure 7). A core set of genes in the regulon appears to be stable (i.e., they are always regulated by *TP53*, no matter the mutation type). The stable genes identified through analysis of CCLE samples match those identified in TCGA samples. However, there are also genes which are differentially regulated depending on the mutation type, potentially driving changes in the biology of the cell. For example, NANOG, a driver of pluripotency,^53^ is repressed by *TP53* and is predicted to be differentially regulated depending on the mutation type. This could drive differences in cancer stem cell traits in tumors. HNF4A is repressed by TP53^54^ in a manner that our data suggests is dependent on mutation type. HNF4A is involved in multiple pathways, and has pro- or anti-tumor effects depending on the cancer type. It can function as an inhibitor epithelial-mesenchymal transition, a key process in cancer progression.^55^ These examples show how the specific somatic mutations of p53 could drive differences in the biology of a tumor.

Mapping the transcriptome of a given sample (and therefore a patient) and studying the effect of the unique observed mutations (or other stress conditions) can pave the way to personalized treatments. Our framework can recover mechanistic inference of gene to gene regulation based on different mutations for each specific sample, or different stress conditions such as hypoxia or irradiation. In this way, biologists and clinicians can further their understanding of pathway regulation in a cause and effect fashion, the epitome of precision medicine.

### Limitations of the study

Our analyses and assumptions have some limitations. The set of downstream targets (regulon) used for our perturbed transcription factor *TP53*, although based on the comprehensive regulon database DoRothEA, is not ground truth in either the included genes or their number. We also consider only the most frequent mutations observed in cancer samples. Including other mutations could re-balance in the numbers in the various categories (different mutations), and could result in better training sets or better predictive scores due to increased sample numbers. Furthermore, although we included in-house RNA-seq experimental validation of perturbations in different stress conditions such as hypoxia and irradiation, further validation of the differential regulation of the mutation types, the deleterious effect they have and the previously identified *TP53* hotspot mutations could be sought. Additionally, the samples analyzed in TCGA are mixed, so while the mutation is only present in some of the cells, the signal comes from all cells. In addition to *TP53*, one may also expand the analysis to different TFs and different diseases. This would further examine the utility of the method, and uncover limitations or adjustments needed for this approach to be generalized. Finally, the computational models on both the ML and the optimization side contain various assumptions and have their own limitations, although both modeling schemes are very well established and polished.

## STAR★Methods

### Key resources table

**Table:** 

REAGENT or RESOURCE	SOURCE	IDENTIFIER
**Deposited data**		

TCGA cancer program	The Cancer Genome Atlas Program	https://www.cancer.gov/ccg/research/genome-sequencing/tcga
CCLE DepMap	Cancer Cell Line Encyclopedia	https://depmap.org/portal/ccle/
cBioPortal	cBioPortal for Cancer Genomics	https://www.cbioportal.org/
TCGA survival data	NIH National Cancer Institute Genomic Data Commons	http://api.gdc.cancer.gov/data/3586c0da-64d0-4b74-a449-5ff4d9136611, https://api.gdc.cancer.gov/data/1b5f413e-a8d1-4d10-92eb-7c4ae739ed81

**Software and algorithms**		

DoRoThEA	DoRothEA: collection of human and mouse regulons	https://saezlab.github.io/dorothea/
Carnival	CAusal Reasoning for Network identification using Integer VALue programming	https://saezlab.github.io/CARNIVAL/
CPLEX	IBM	https://www.ibm.com/products/ilog-cplex-optimization-studio/cplex-optimizer
XGBoost	*R* Statistical Language	https://cran.r-project.org/web/packages/xgboost/index.html
Elastic-net	*R* Statistical Language	https://data.mendeley.com/datasets/rn96hp5kw4/2

### Resource availability

#### Lead contact

Further information and requests for resources and reagents should be directed to and will be fulfilled by the Lead Contact, Francesca M. Buffa (francesca.buffa@unibocconi.it, francesca.buffa@oncology.ox.ac.uk).

#### Materials availability

This study did not generate new unique reagents.

#### Data and code availability

Any additional information required to reanalyze the data reported in this paper is available from the lead contact upon request. The implementation and the input data with the full results are available as a Shiny app developed in *R* (v.4.2.2) at https://data.mendeley.com/datasets/rn96hp5kw4/2. Additionally, all required code can be found at our GitHub repository here.

### Method details

We combined multiple computational techniques and modeling schemes to provide an integrated platform for rapidly performing experiments given CCLE or TCGA datasets. We extracted the predicted regulon from DoRothEA. The regulon itself adapts to the optimization output each time; given the initial network of genes and their known interactions, some may be dismissed as nodes or their edge type (mode of regulation) might be altered to fit the *expression (training)* data, which contain the gene expression profiles. The input data is usually perturbation simulations, where deleterious mutations observed in *TP53* are assumed to render the protein dysfunctional, allowing the model to predict the regulon using optimization to best fit the gene expression data. The inference achieved in this way can serve as a basis to create a network that allows us to study the downstream effects of specific p53 mutations.

Once the signature and the expression matrix are selected, CARNIVAL attempts to optimize the selected PKN network by using information from the RNAseq expression values, the PKN and the measurements for the transcription factors included in the initial network (Normalized Enrichment Scores as those extracted from the R package VIPER). The resulting optimized network assigns states (node-wise) and relationships (edge-wise). More specifically, the TF will be marked as the perturbation node, and downstream of this the nodes (genes/proteins) are either up-regulated (red) or down-regulated (blue) with the corresponding edges acting either as an activator or inhibitor, from source to target (the PKN is directed). Each optimized network is stored and characterized by the single *TP53* mutation it corresponds to. We then computationally compare all networks across each other. Comparisons are being calculated on the basis of four different settings: i) general, such as we do not filter the compared networks across any *TP53* feature, ii) same type of mutation, iii) deleterious attribute of *TP53* mutation, and finally iv) mutations that have been characterized as known hotspots for *TP53*.

#### Cancer Cell Line Encyclopedia (CCLE)

We used the Cancer Cell Line Encyclopedia omic datasets,^56^ *DepMap Public* to train the networks. We collected all the cell lines corresponding to each of the 22 different cancer types. The expression profiles serve as an input to both the linear regression and the CARNIVAL optimization modules. The mutational profiles are used for CARNIVAL to extract mutational status and further features (such as deleterious function) for *TP53*, which is the perturbed input in the network for all optimizations performed.

#### The Cancer Genome Atlas (TCGA)

We used the survival data as provided on the NIH National Cancer Institute Genomic Data Commons website respectively (see key resources table). We then combined the expression profiles with mutations downloaded from *cBioPortal*. This provides the full input for either the Machine Learning pipeline and CARNIVAL experiments. We extract the same semantic fields such as deleterious function, as initially described for the CCLE datasets.

#### Radiation experiment

H460 (p53 wild-type Non-small cell lung cancer) cells were grown in parallel in two 175 cm2 flasks with RPMI-1640 media supplemented with Fetal Bovine Serum (10%), Penicillin-Streptomycin (1%) and L-glutamine (1%). Media was changed three times per week and passaged with trypsin (1%) on approaching confluency using aseptic technique. A cobalt gamma irradiator was used to deliver 50/60 Gy in 2 Gy per fraction over 5/6 weeks. Cell lines were authenticated via genomic analysis (Northgene) and underwent regular mycoplasma testing. Following irradiation sub-lines were confirmed to be radio-resistant versus parental via clonogenic assay. An RNAseq experiment was performed for the non-irradiated parental (H460 BASE) and sublines (H460-60A and H460-50B) at 5 time points; pre-(0 hrs) and post-(2, 6, 12 and 24 hrs) a further 2 Gy irradiation. Cells were seeded to 6 well plates the prior day and harvested per timepoint, three biological replicates were performed per condition and total RNA extracted using TriZol (Thermofisher) following manufacturer’s protocol. Library preparation was performed with Lexogen Quantseq 3′ RNA kit and sequencing with the Illumina NovaSeq platform by Welcome Trust Center for Human Genetics Oxford (WTCHG).

#### Hypoxia experiment

##### Cell culture

All cell lines used (MCF7 Cat HTB-22 RRID:CVCL _ 0031; MDA-MB-231 Cat CRM-HTB-26 RRID:CVCL _ 0062; MDA-MB-453 Cat HTB-131 RRID:CVCL _ 0418; MDA-MB-468 Cat HTB-132 RRID:CVCL _ 0419; and HCC1806 CatCRL-2335 RRID:CVCL _ 1258) were purchased from ATCC. They were routinely cultured in DMEM low glucose (1 g/L) and supplemented with 10% FBS no longer than 20 passages They were mycoplasma tested every 3 months and authenticated during the course of this project. For the larger experiment, cells were cultured in different glucose (Gluc) and glutamine (Gln) levels as follows: medium A) 1 mM Gln, 5 mM Gluc; medium C) 4 mM Gln, 5 mM Gluc; medium D) 1 mM Gln, 2 mM Gluc; and medium F) 4 mM Gln, 2 mM Gluc; and subjected to normoxia (21%), 1% or 0.1% hypoxia for 24 h and 48 h using an InVivO2 chamber (Baker). For the smaller experiment, cells were cultured in either: medium C, DMEM high glucose (4.5 g/L) 4 mM Gln, Human Plasma Like Medium (HPLM; Gibco A4899101), or Plasmax.^57^ All media were supplemented with 10% FBS. Cells were seeded the day prior to the experiment, then cultured under normoxia (21%) or hypoxia (1%) for 24 h.

##### RNA extraction and DNAse treatment

RNA from each experiment was extracted using the mirVanamiRNA Isolation Kit (AM1560, Thermo Fisher Scientific) and DNAse treated with TURBO DNA-freeKit (AM1907) following the manufacturer’s instructions.

#### Survival models

We used the Kaplan-Meier(K-M) model in *R* using the *survival* package. The model visualisation was done using *R* package *survminer* and the function *ggsurvplot*, enabling the display of automatic calculation for the log rank test for the p value. Features *OS.time, OS* were extracted from the TCGA survival data to perform the analyses.

#### Elastic-net and XGBoost classifiers

The elastic-net models are obtained via an in-house implemented *R* package developed to obtain reliable estimates of the true error of a trained model, i.e., the difference between the true value and the approximation resulting from the model prediction. The estimate of the true error is important as it allows us to understand how well the developed model generalised to unseen data. Our software does this by using a multiple sampling strategy that can be summarised in the pseudo-code below:

**Data:** Choose sampling strategy and learning methods & create a grid of allowed training set sizes based on previous choices

**for** each training set size **do**
  **for** each sampling iteration **do**
    sample a training set and test settune the hyper-parameters fit a model on training set predict a model on test set compute an accuracy (model) measure
  end for
end for

Choose the optimal model For each size, data were randomly split into training and test sets using a stratified random sampling approach: the training data was used to fit a generalised linear model with L1/L2 penalisation (elastic-net), while the test set was used to assess its performance. The hyper-parameters of the elastic-net models (i.e., alpha and lambda) were selected from a grid of provided values via 10-fold cross-validation in order to obtain the minimum mean cross-validated error. After the hyper-parameters were fixed, the final model was fitted on the entire training set and tested on the left-out data. The above steps were repeated for multiple random samples of the data, in order to estimate the mean error of our procedure and the related 95% confidence interval (CI). Finally, an overall best model was selected by firstly choosing the optimal training set size, i.e., the size showing the lowest upper bound of the 95% CI, and then by identifying the model with the minimal misclassification error across all the trained models built using such size. The CI of the mean error for the i-th training set size is defined as:

$$CI_{i}=M_{i}\pm c\times SEM_{i}$$

where *c* is the *critical value* and depends on the desired confidence level (our default value is 95% confidence level), and $SEM_{i}$ is the standard error of the mean error. $SEM_{i}$ is computed as:

$$SEM_{i}=\frac{standard\;deviation\;of\;errors_{i}}{\sqrt{n}}=\frac{s_{i}}{\sqrt{n}}$$

The sample standard deviation of the performance metrics $\left(s_{i}\right)$ is calculated as the squared root of the sample variance using the unbiased estimator:

$$Var_{i}=s_{i}^{2}=\frac{1}{n-1}\sum_{j=1}^{n}{\left(error_{ij}-M_{i}\right)}^{2}$$

So, the $SEM_{i}$ is:

$$SEM_{i}=\sqrt{\frac{1}{n\left(n-1\right)}\sum_{j=1}^{n}{\left(error_{ij}-M_{i}\right)}^{2}}$$

The sampling strategy used in this work is multiple random sampling, while the used learning method is GLM via elastic net regularisation. A feature screening was performed inside cross-validation to reduce the high-dimensionality of the feature space and select the variables most strongly related with the response. The package has been developed by Dr Alessandro Barberis and Prof Francesca M. Buffa at the department of Oncology, University of Oxford, UK. For the XGBoost classifier, we used the *XGboost* package in *R*, with a non-linear *softmax* objective function for multi-label classification. Labels were defined on the basis of the number of different unique mutation types in CCLE (7) and TCGA (10) for *TP53*. The classifier was applied on all samples (pancancer) per database (CCLE and TCGA separately) and we used a 70/30 split training/test set. Parameters and output in detail can be found in the supplementary information.

#### Optimization/reconstruction of networks

CARNIVAL^58^ uses Mixed-Integer Linear Programming to optimize/train a given Prior Knowledge Network (PKN) which serves as a starting point on how the topology of the interactions is delineated. For a given single condition, the model then fits the experimental data (expression) for the nodes of the network altering either the type of the interaction or dropping/adding a node (gene-protein) as the size of the network is penalized given the NES activities. The resulting optimized network minimizes the mismatch between the measurements and the predicted states (up or down regulation) of the network nodes. In this way, a single mutation of *TP53* mapped as a unique condition and a perturbation input for our network can be assessed for its downstream impact on the regulon by comparing the two networks. Effectively, we then stratify the comparison across the same settings we categorized earlier the binomial classifiers: i) mutation type, ii) deleterious function and iii) *TP53* hotspot mutations. We then analyze how strong of a signal we receive for low, medium and high/very high similar networks (correspondingly 25, 50, 75 and above 90% similarity scores) to assess which of the settings analyzed explains a similar downstream effect for each *TP53* mutation/condition.

#### Network comparison

Let $G_{A},G_{B}$ be the two under comparison re-constructed (optimized) networks. Let $G_{A}=\left\{E_{A},V_{A}\right\}$ and $G_{B}=\left\{E_{B},V_{B}\right\}$ be the sets of edges and vertices correspondingly for the two networks as graph representations. The intersection of the two networks is the set of edges that exist in both networks. More formally, if *G* is the intersection of $G_{A},G_{B}$:

$$G=graph.intersection\left(G_{A},G_{B}\right)=\left\{E,V\right\}:E_{i}\in E_{A}\cap E_{B}\;i\in \left|E\right|\text{.}$$

Without loss of generality, $\left|G_{A}\right|\geq \left|G_{B}\right|$, where |G| is the size (number of edges) of graph *G*. Then, a score of similarity $S_{AB}$ can be calculated between $G_{A},G_{B}$ as follows:

$$S_{AB}=\frac{\left|graph.intersection\left(G_{A},G_{B}\right)\right|}{\left|G_{A}\right|},S_{AB}\in \left[0,1\right]$$

This intersection takes into account node-to-node both direction and type of interaction, therefore yielding a good estimate of how close (or far apart) semantically the compared networks are. The idea of comparing the resulting graphs for similarity we introduce here has also been used in a similar way in the DCI algorithm,^59^ where edges that appeared/disappeared or changed weight are assessed for inference in contrasting two different conditions from the trained respective networks. Network similarity measures have also been used in disease-gene association studies before.^60^ To compare the networks computationally, we create a matrix with each network as a row and column and then take all possible different comparisons. We then exploit the structure of the matrix (symmetric) and efficiently compute all similarity scores. This procedure is systematically done across all optimized generated networks in both CCLE and TCGA datasets.

#### Optimization model

The CARNIVAL optimization model is a Mixed-Integer Linear Programming (MILP) model. It is a special case of constrained optimization where we try to optimize (minimize or maximize) a linear function over a set of also linear constraints under the additional constraint that a subset of our variables have to be real or integers (or just binary x∈{0,1}). This belongs to the general family of Linear Programming optimization models, for which deterministic optimal algorithms exist. For this purpose we used the IBM CPLEX MILP solver.

The objective function attempts to predict the status of the optimized nodes in the network as those up-regulated (1) or down-regulated (−1) which has to abide by the so-called *consistency rules* which enforce biologically reasonable interactions between the genes. This becomes an optimization problem as we force to minimize the function over a feasible set of constraints. The full specifications on the semantics, derivation, and explanation of the mathematical constraints in detail can be found in.^58^

### Quantification and statistical analysis

All statistical analyses were performed in *R* v.4.2.2. Statistical tests were performed within the stat_compare_means function and included T-test, Wilcoxon, Kruskal-Wallis and Anova tests (significance level 5%). For the survival models log rank tests were performed in the *survminer* (version 0.4.9) using the *ggsurvplot* function.
