# Supplementary material for: A machine learning and directed network optimization approach to uncover TP53 regulatory patterns
Source: iScience. 2023 Oct 26;26(12):108291. doi: 10.1016/j.isci.2023.108291 (PMC10692668; doi:10.1016/j.isci.2023.108291)
Supplement: Document S1. Figures S1–S22, Tables S1, and S2 [file mmc1.pdf]

iScience, Volume *26*

Supplemental information

A machine learning and directed network optimization approach to uncover *TP53* regulatory patterns

Charalampos P. Triantafyllidis, Alessandro Barberis, Fiona Hartley, Ana Miar Cuervo, Enio Gjerga, Philip Charlton, Linda van Bijsterveldt, Julio Saez Rodriguez, and Francesca M. Buffa

Supplemental Information

A machine learning and optimization approach to uncover TP53 regulatory patterns

Charalampos P. Triantafyllidis, Alessandro Barberis, Fiona Hartley, Ana Miar Cuervo, Enio Gjerga, Philip Charlton, Linda Van Bijsterveldt, Julio Saez Rodriguez, Francesca M. Buffa

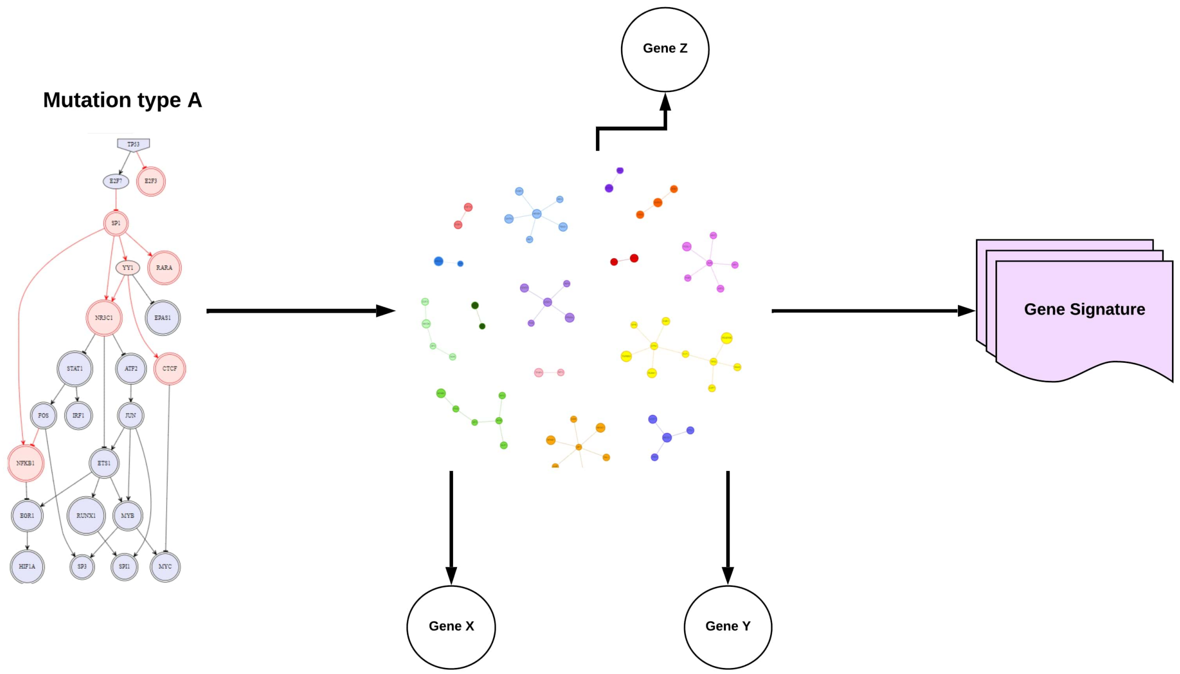

Supplemental Figure 1: The methodological process to extract gene signatures per mutation from the optimized and reconstructed networks. On the left we see an optimized network which corresponds to a specific mutation type (A) for *TP53*. This network is then partitioned using community detection (Louvain Method) and then each community is mapped to a single gene (as shown, X, Y and Z.) by the maximum betweeness centrality score (red node). These genes are then merged and form the gene signature for the specific network. Then, taking the overlap of all these gene signatures extracted from all networks that correspond to the same mutation type of *TP53*, provide the meta-signature for this mutation type. This process has been repeated across both CCLE and TCGA data sets.

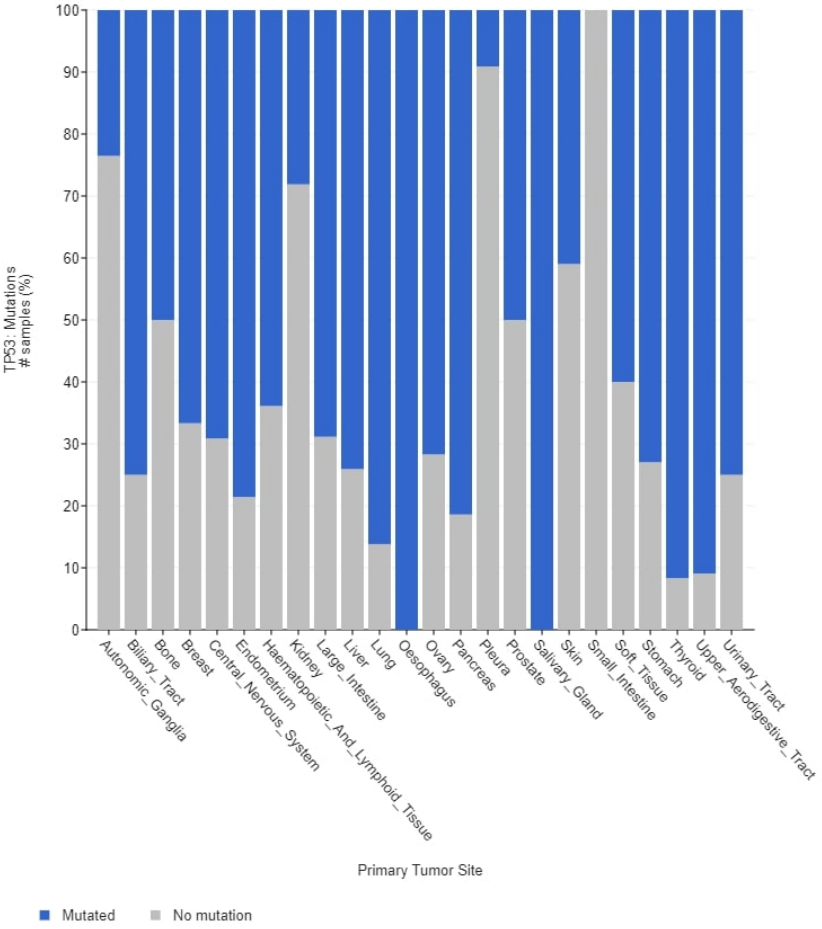

(a)

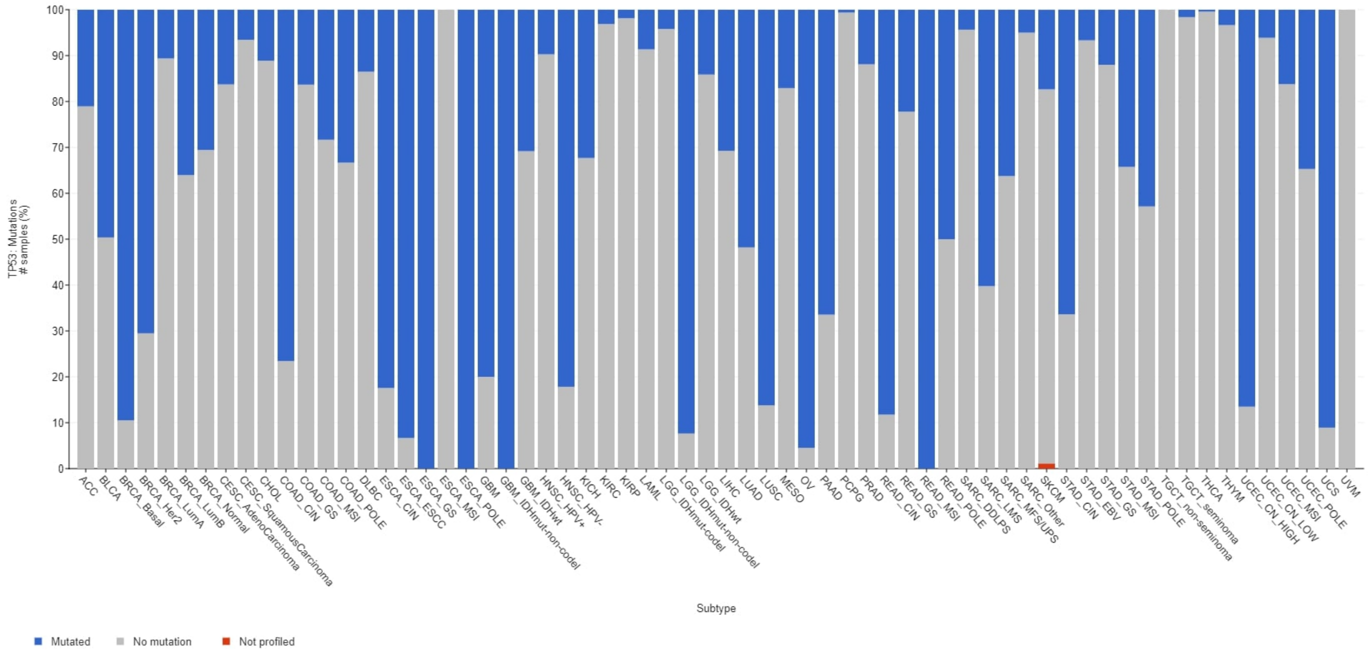

(b)

Supplemental Figure 2: Mutational frequency of *TP53* per primary site in CCLE (a) and sub-type in TCGA (b) (source : `https://www.cbioportal.org/`).

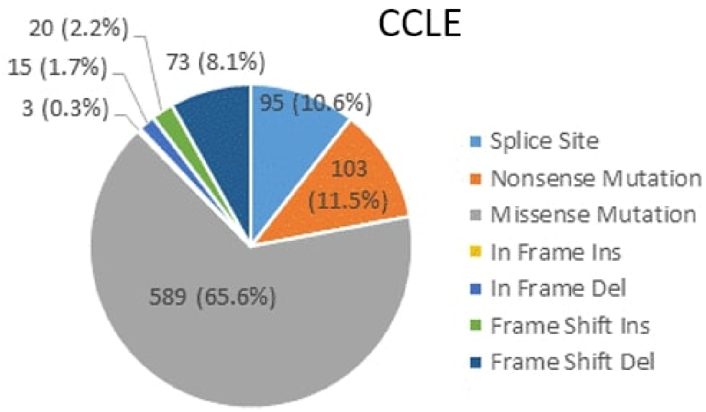

(a)

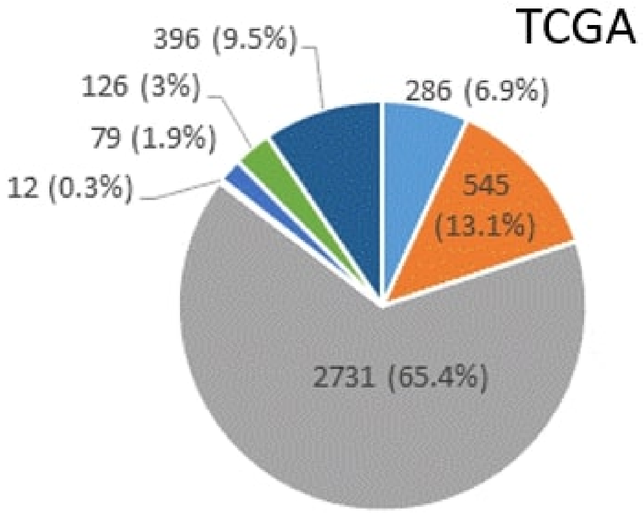

(b)

Supplemental Figure 3: a) *TP53* mutational variation count in DepMap CCLE v.20Q2. A total of 898 cell line samples with a *TP53* mutation were identified. Ten cell lines had more than one *TP53* mutation. b) *TP53* mutational variation count in TCGA. A total of 4250 tumour samples with a *TP53* mutation were identified.

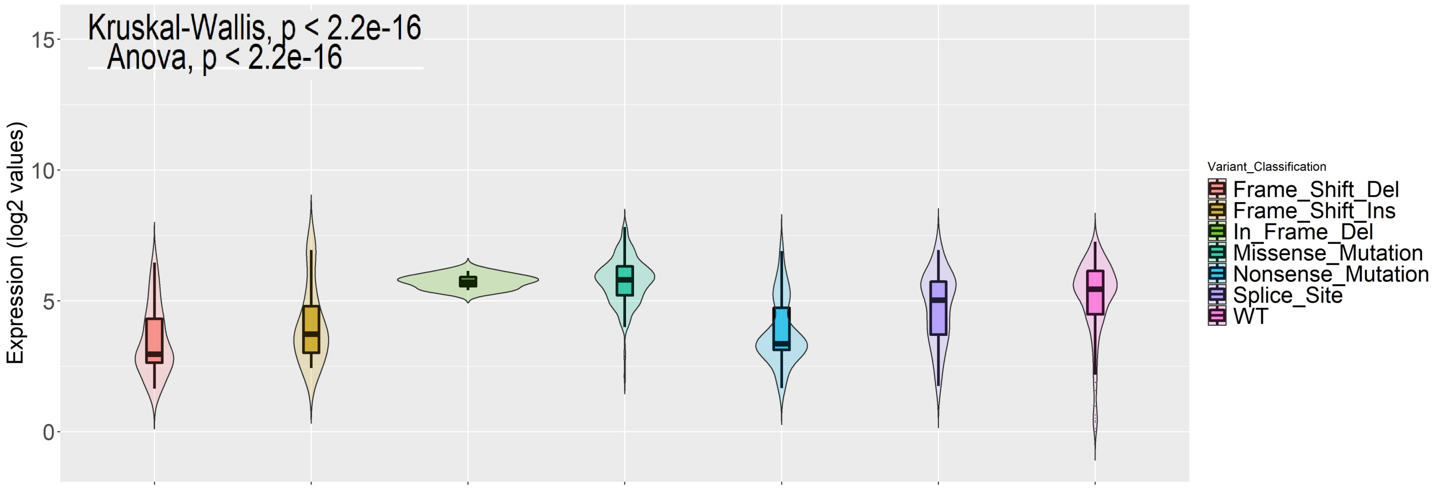

(a)

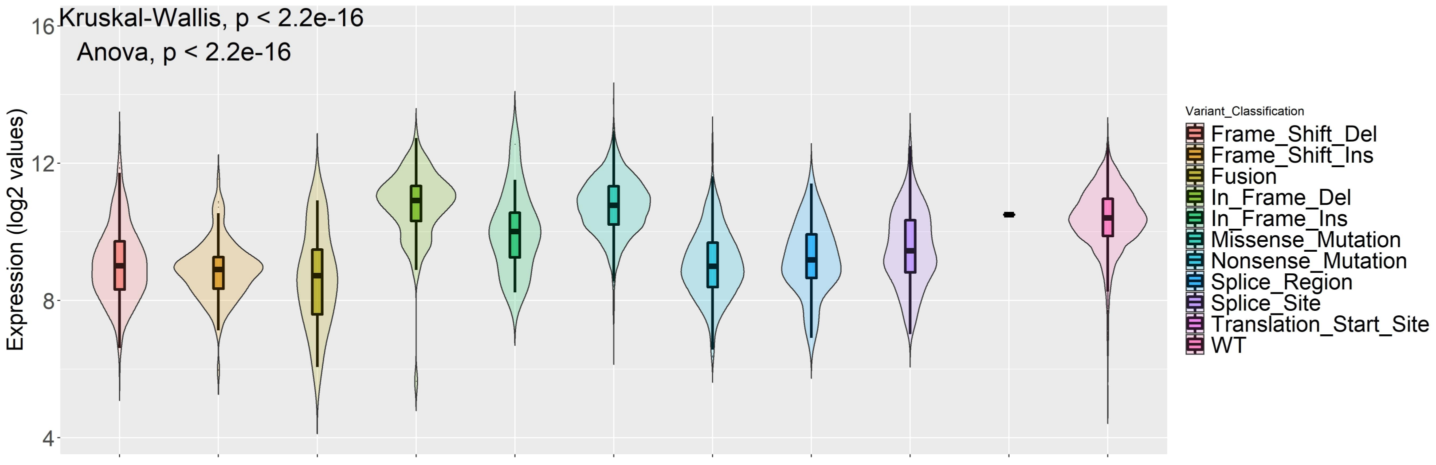

(b)

Supplemental Figure 4: Association between mutations and expression (RNAseq) of *TP53* in CCLE and TCGA data. Sources: TCGA PanCancer Atlas and DepMap Public 21Q2.

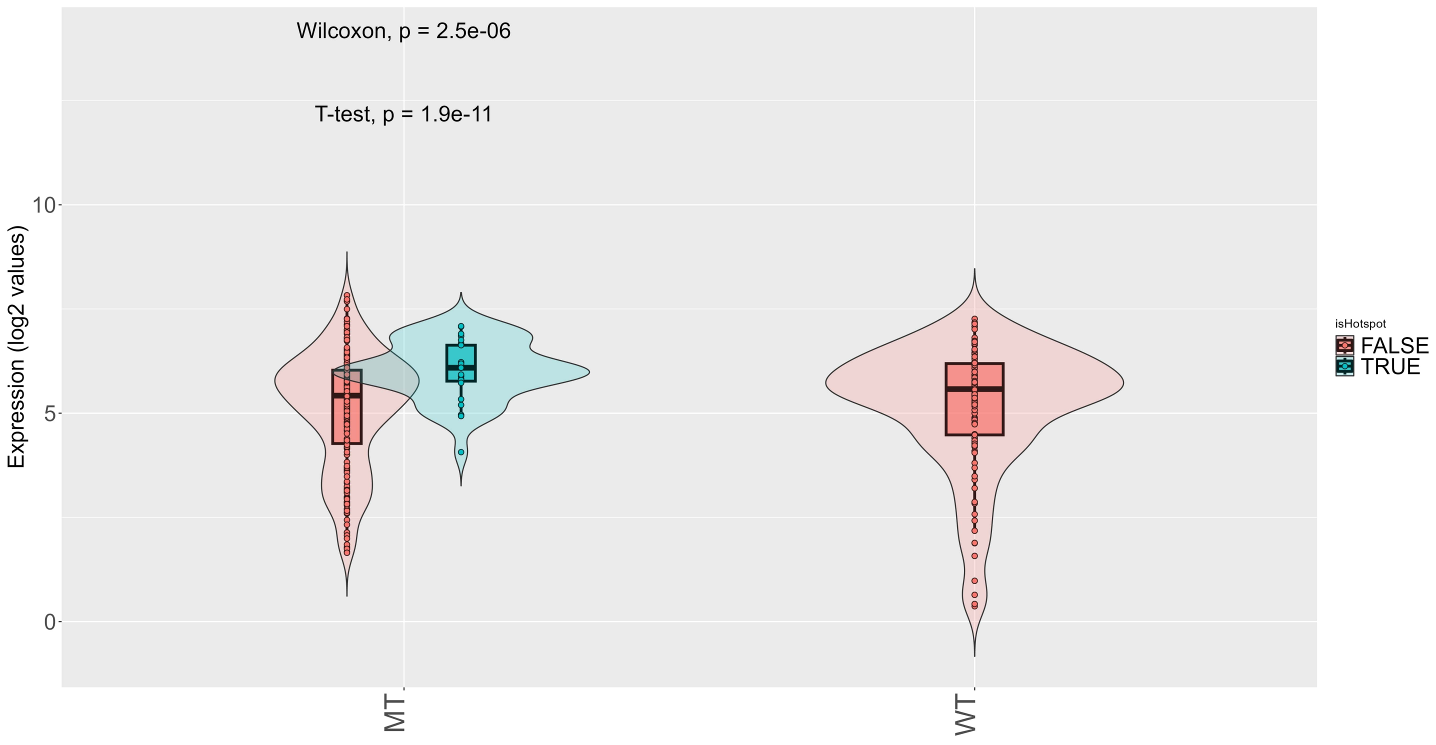

(a)

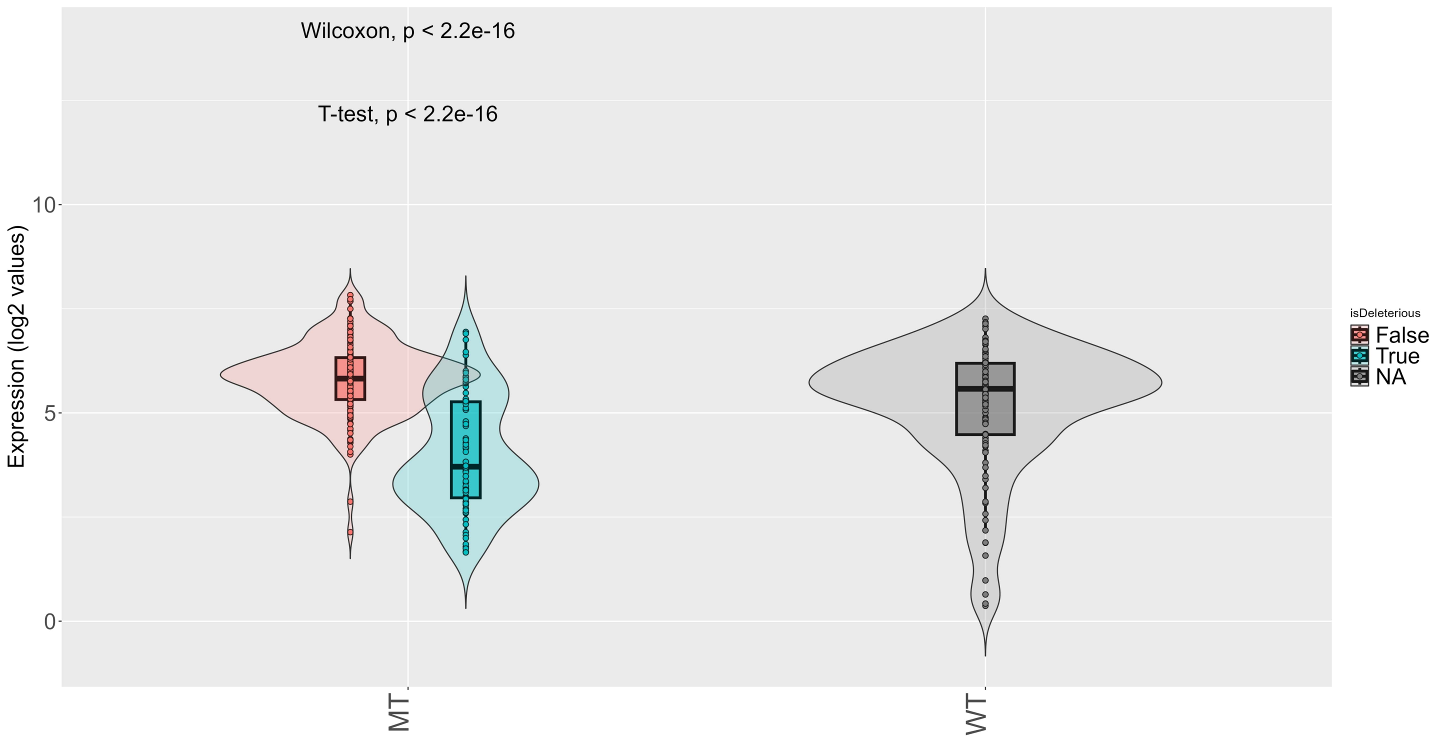

(b)

Supplemental Figure 5: 5(a) CCLE: Hotspot versus non-hotspot (p.R175H, p.R248Q, p.R273H, p.R248W, p.R273C, p.R282W, p.G245S) mRNA expression between mutated and WT samples and 5(b) Deleterious versus non-deleterious mRNA expression of *TP53* between mutated and WT samples.

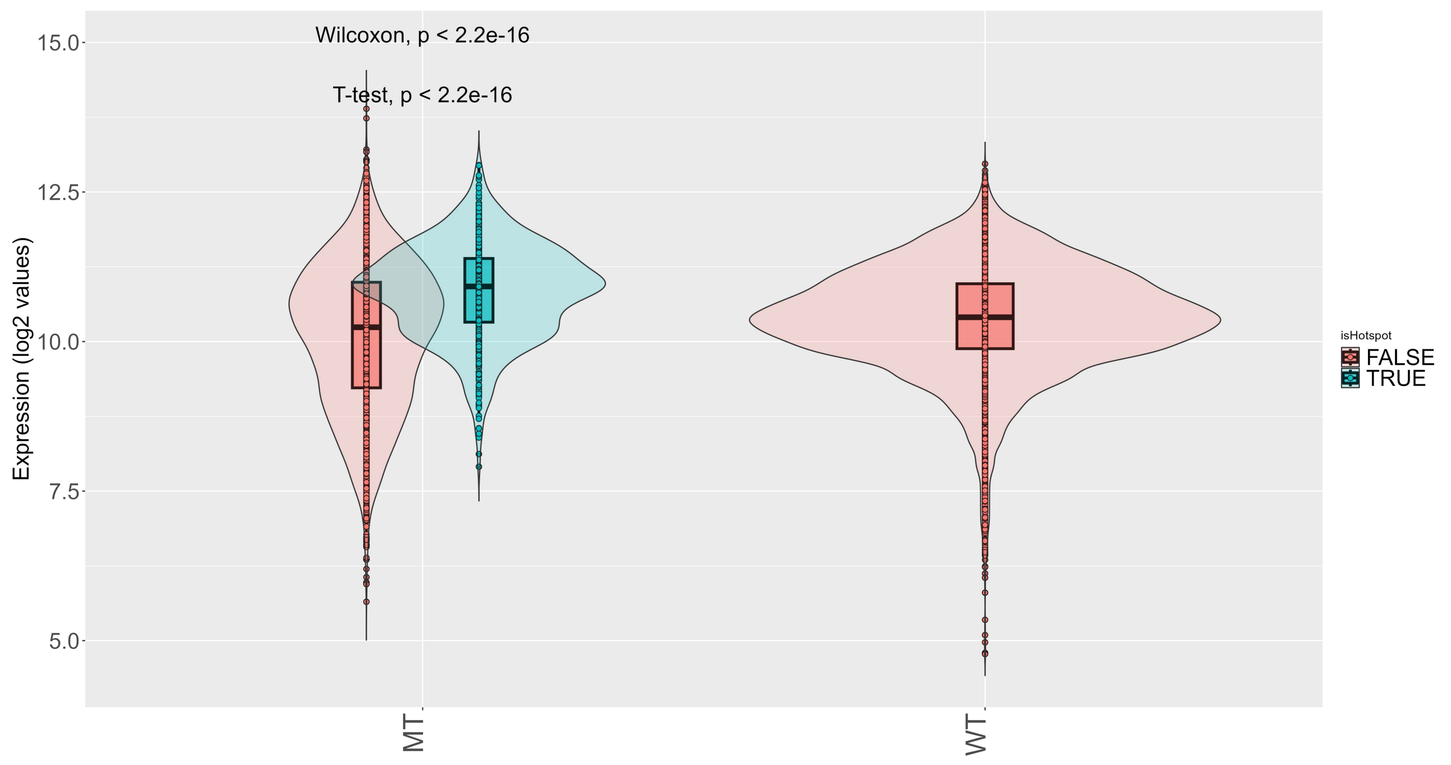

(a)

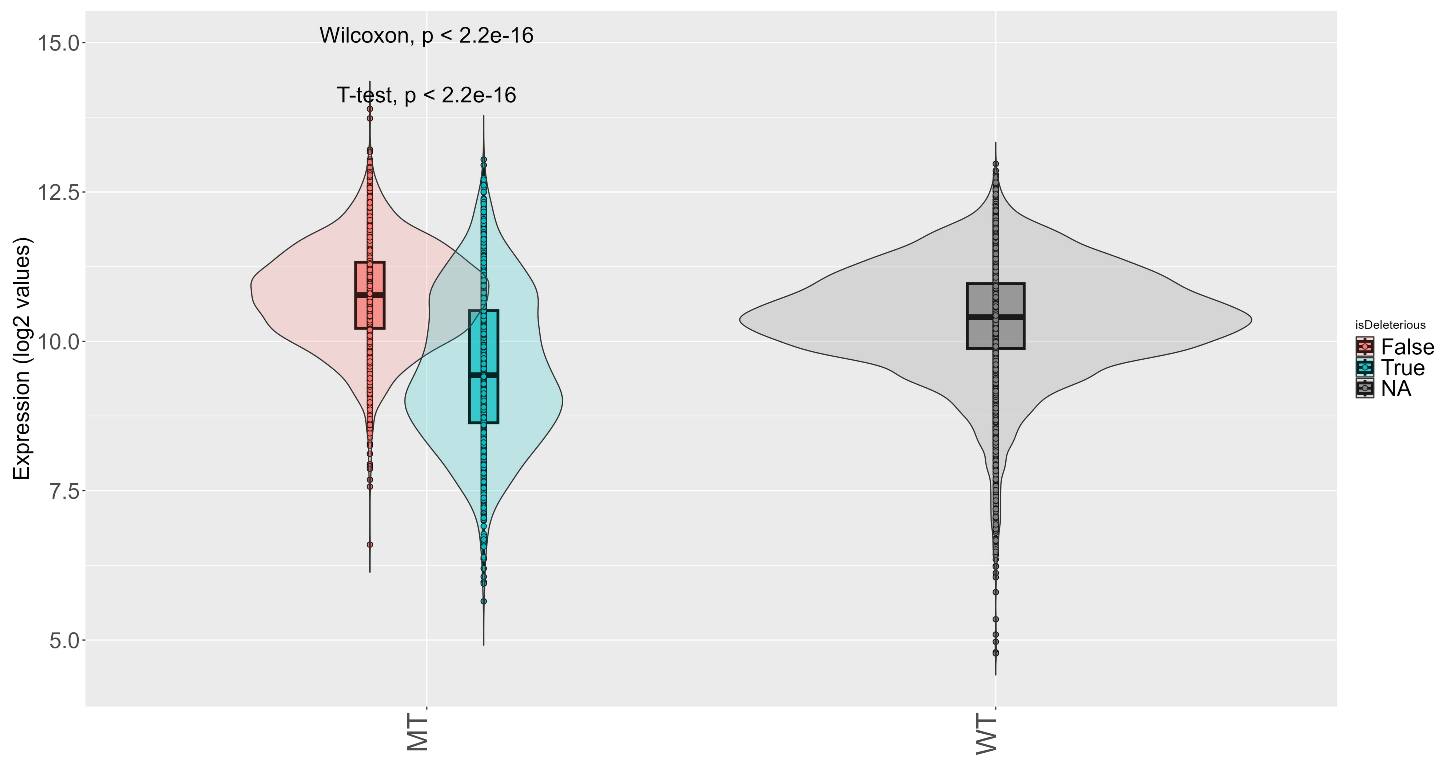

(b)

Supplemental Figure 6: 6(a) TCGA: Hotspot versus non-hotspot (R175H,R248Q,R273H,R248W,R273C,R282W,G245S) mRNA expression between mutated and WT samples and and 6(b) Deleterious versus non-deleterious mRNA expression of *TP53* between mutated and WT samples.

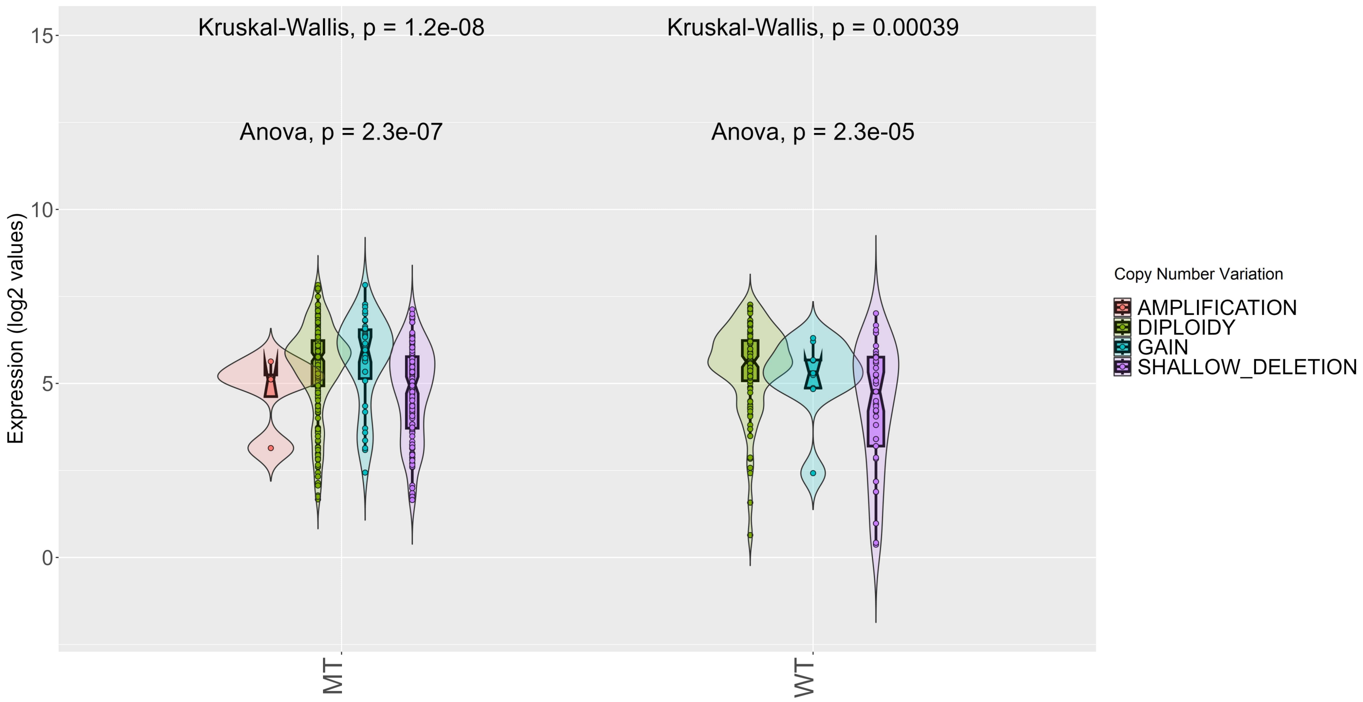

(a)

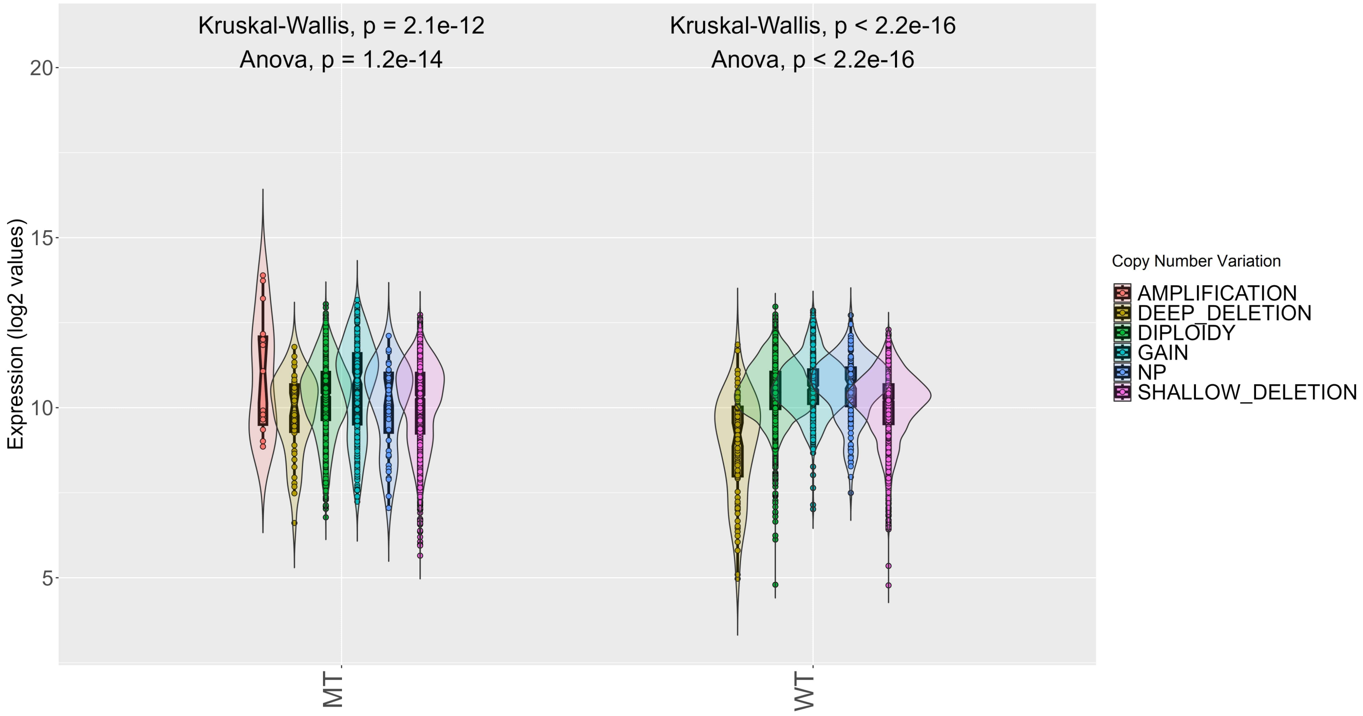

(b)

Supplemental Figure 7: Violin plots (distribution and boxplots of mean 95% confidence interval within each notch) of *TP53* expression (mRNA) in both mutated (MT) and Wild-type (WT) samples versus copy number alternation (CNA) in both CCLE (a) and TCGA (b) databases, calculated using the GISTIC algorithm (*Genomic Identification of Significant Targets in Cancer* (GISTIC)[?]) and categorized as follows: *Deep Deletion* indicates a deep loss and possibly a homozygous deletion, *Shallow Deletion* indicates a shallow loss, *NP* (no alteration), *Diploidy*, *Gain* indicates a low-level gain (a few additional copies, often broad), *Amplification* indicates a high-level amplification (more copies, often focal) - source : `https://www.cbioportal.org/)`.

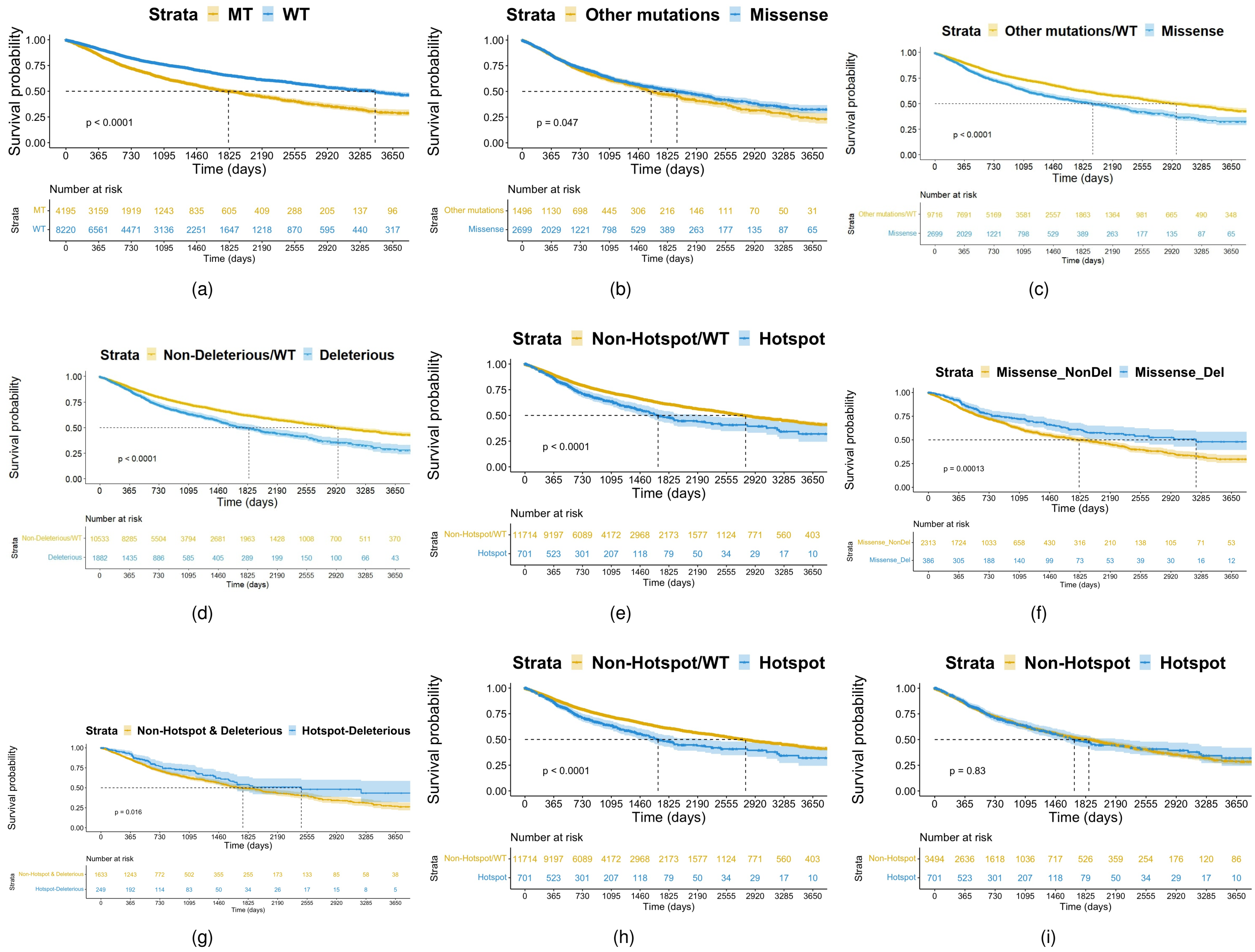

Supplemental Figure 8: Using the TCGA survival data to plot survival curves on various binomial settings of *TP53* status: i) MT versus WT 8(a) (p<0.0001) , ii) All mutations versus missense 8(b) (p=0.047), iii) All mutations and WT samples versus Missense mutations 8(c) (p<0.0001), iv) Non deleterious mutations and WT samples versus Deleterious mutations 8(d) (p<0.0001), v) Non-Hotspot Mutations and WT samples versus Hotspot mutations 8(e) (p<0.0001), vi) Missense Non Deleterious mutations versus Missense Deleterious 8(f) (p<0.00013) and vii) Non Hotspot deleterious mutations versus Hotspot Deleterious 8(g) (p=0.0016). The figures show that certain features such as missense mutations affect survival probabilities significantly. Additionally, survival curves for Hotspot mutations of *TP53* versus every other mutation 8(h) (p<0.0001) (including WT samples) and 8(i) (p=0.83) Hotspot mutations of *TP53* versus every other mutation (not including WT samples) are given.

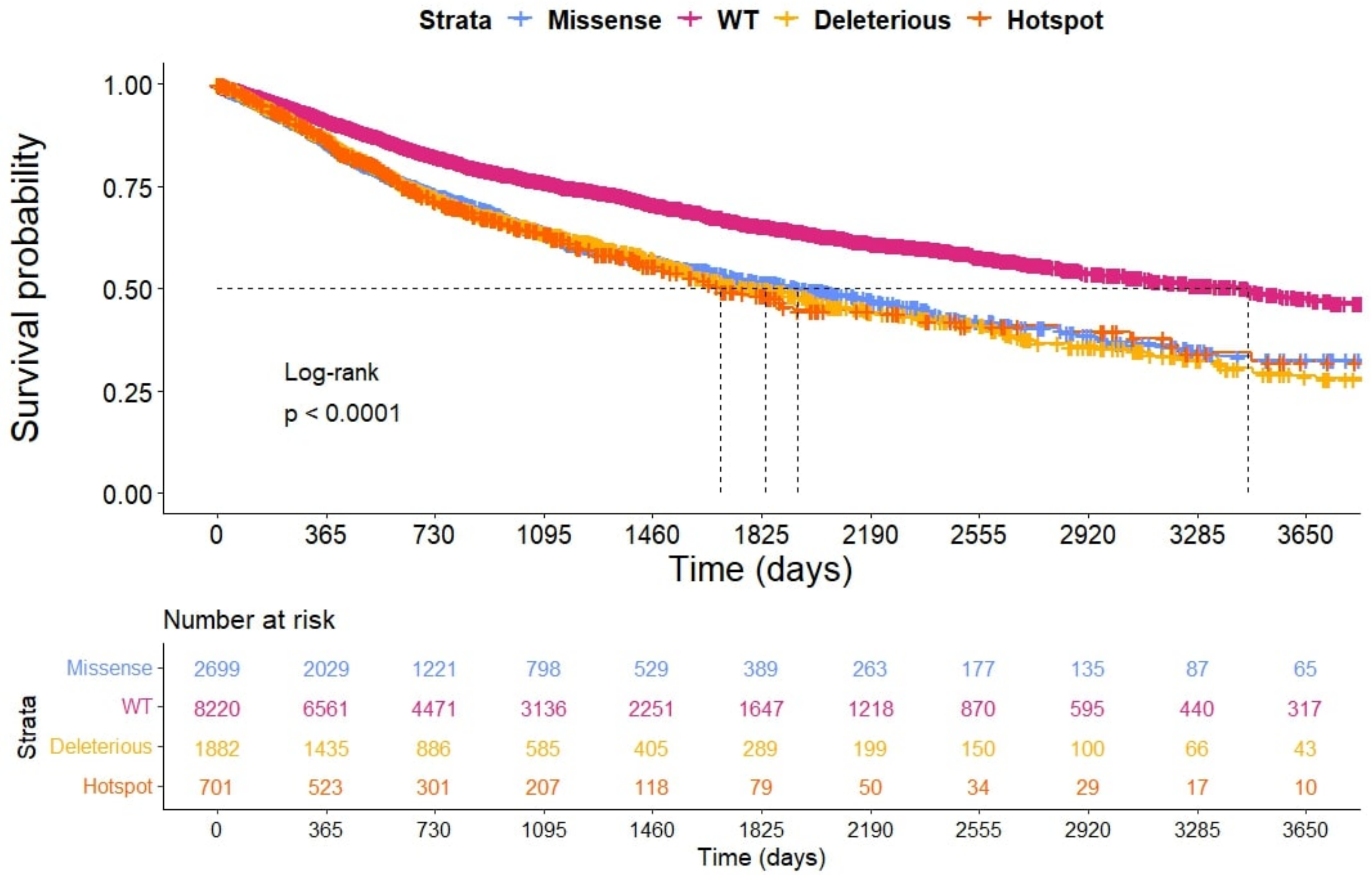

Supplemental Figure 9: Kaplan-Meier curve on survival data from TCGA, plotting the curves corresponding to the following mutational features for *TP53*: i) Missense mutations (blue), ii) WT samples (magenda), iii) Deleterious mutations (yellow) and iv) Hotspot mutations (orange).

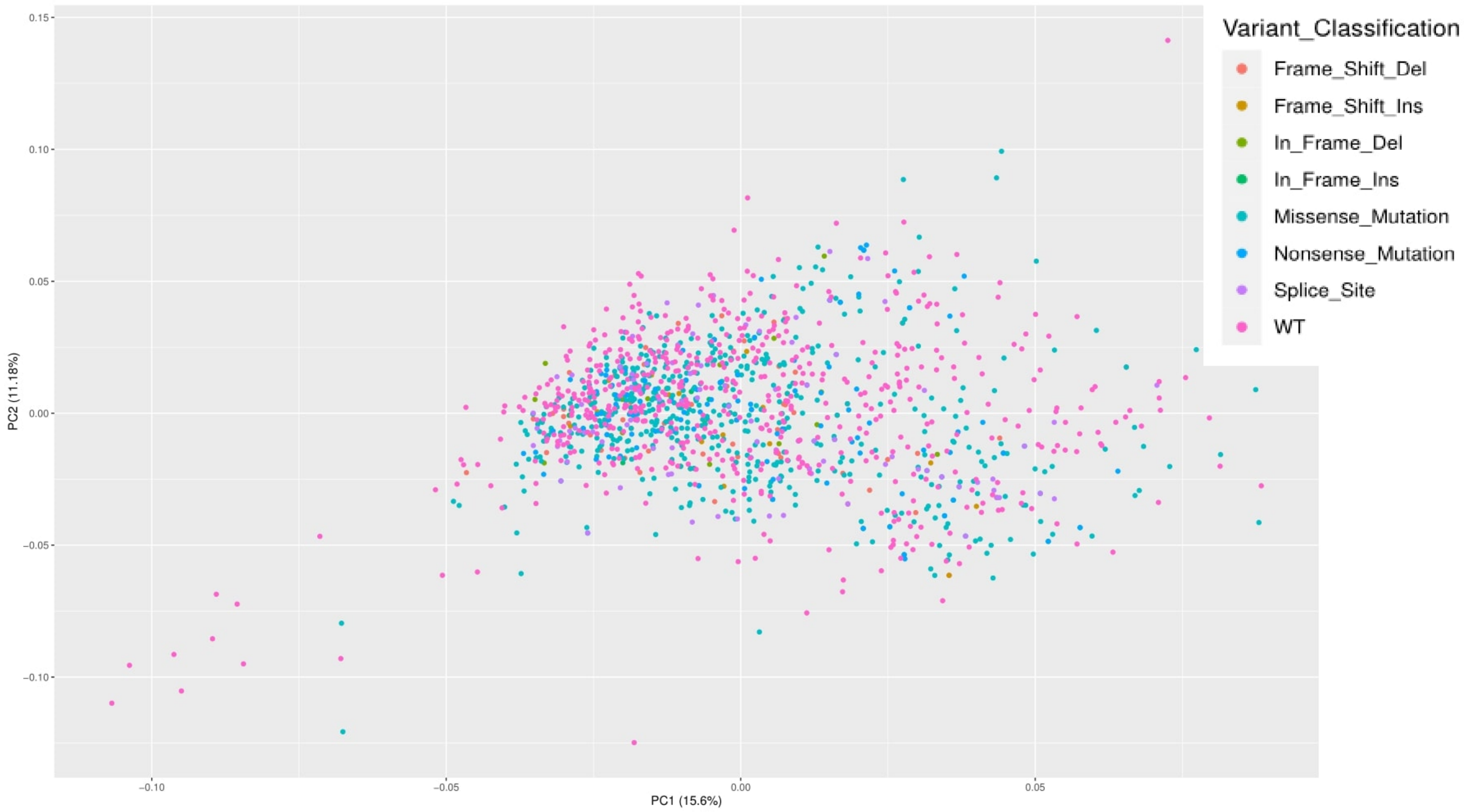

Supplemental Figure 10: Principal component analysis (PCA) of the expression (RNAseq) of the regulon of *TP53* in cell lines samples (CCLE). Principal component 1 (PC1) and 2 (PC2) are shown with the associated variability.

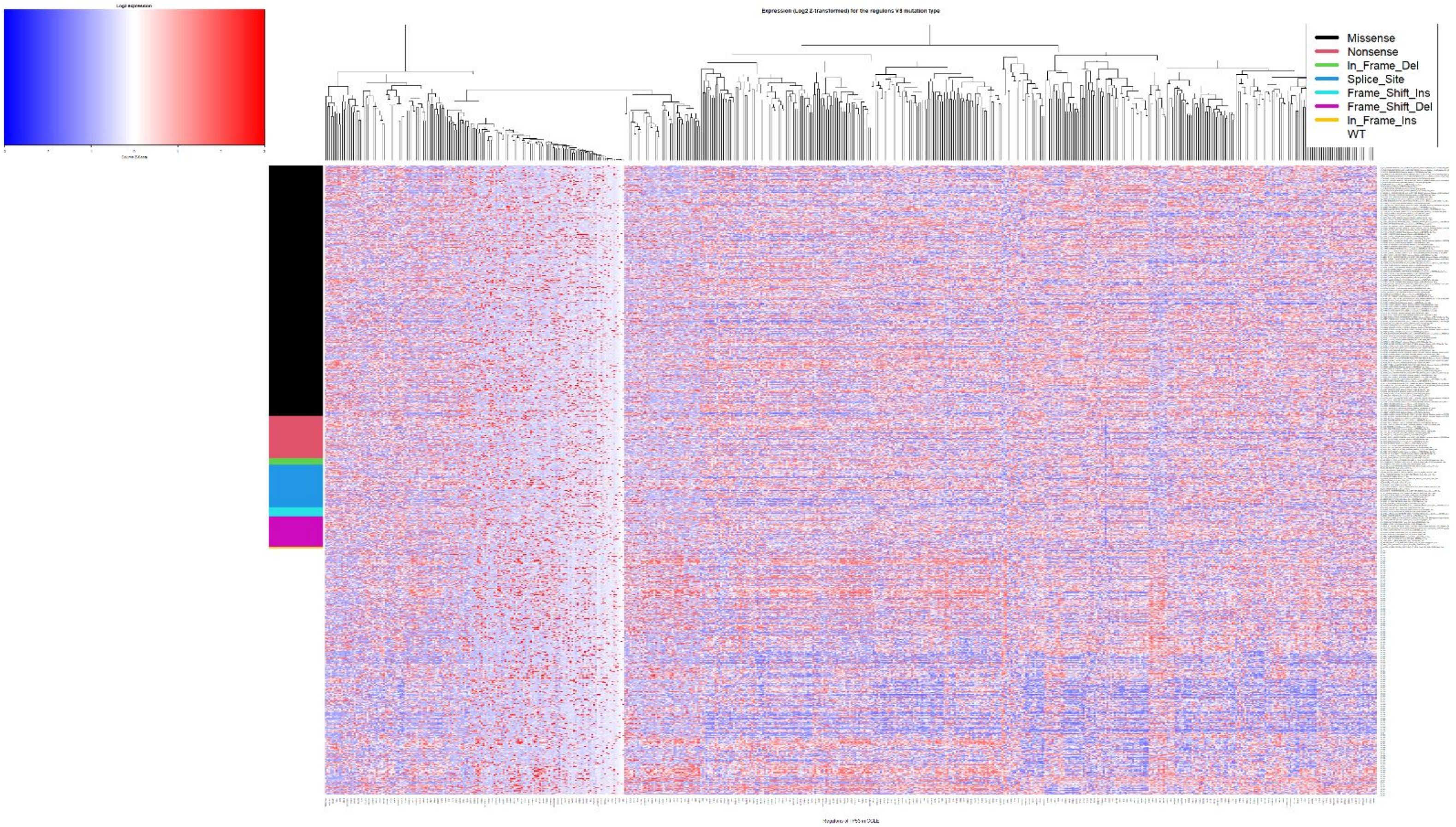

Supplemental Figure 11: Heat-map of expression (log2) in CCLE for *TP53* across all cell lines versus different mutation types and WT samples. Each mutation type appears with a different color in the legend, and are sorted to be easily differentiated as groups. The white (not colored) are the WT samples.

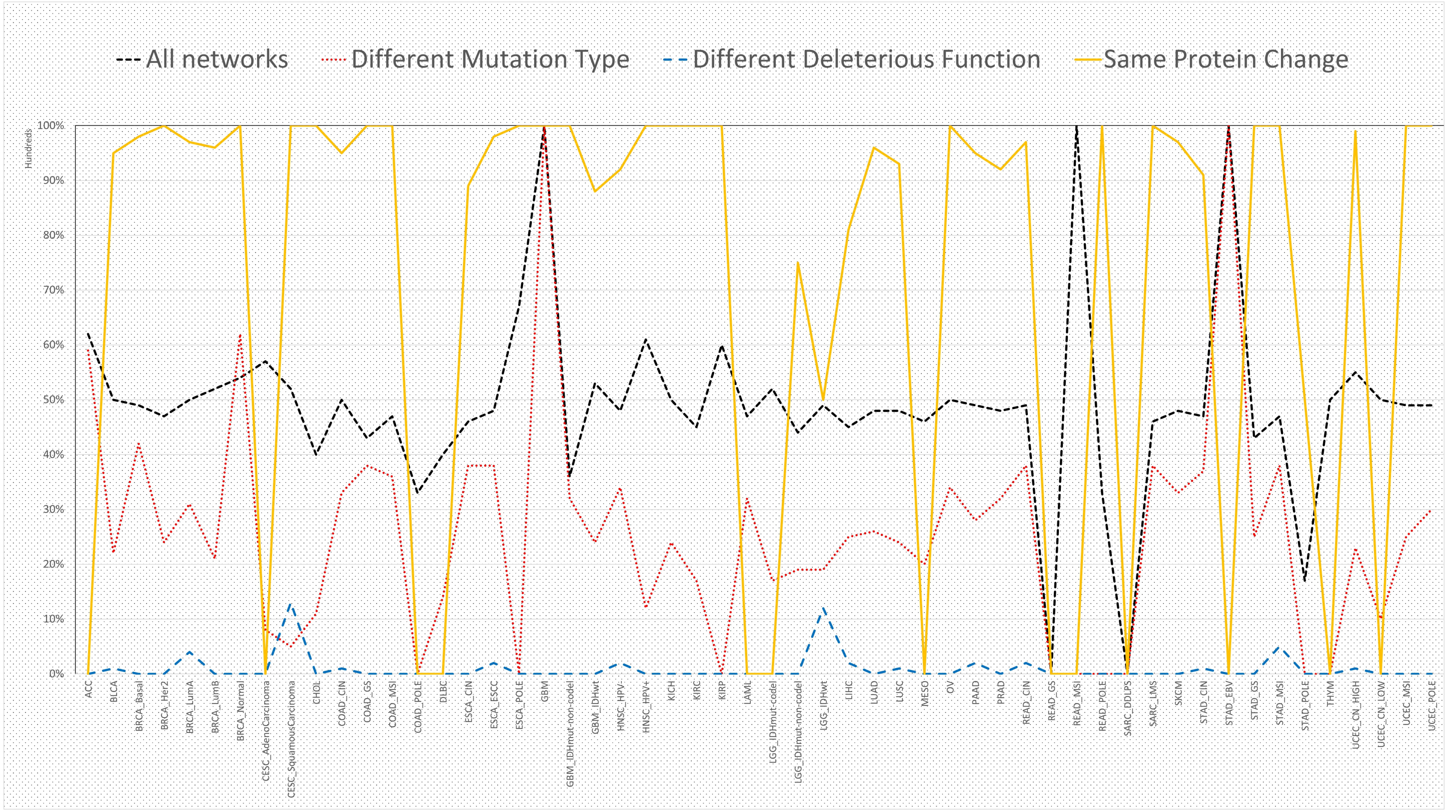

Supplemental Figure 12: A different aspect of the computational study presented in this figure, using TCGA data (x- axis for cancer types and y-axis for percentage of similarity; using a 50% similarity cut-off, we stratify only across i) all networks, ii) different mutation types, iii) different deleterious function (deleterious versus non-deleterious compared only) and iv) same protein change. We observe how similarity diminishes in the case where we compare different mutation type or deleterious function of mutation, as expected based on our previous results, whereas same protein change produces similar networks.

	CCLE Cancer	G25	G50	G75	G90	M25	M50	M75	M90	D25	D50	D75	D90	H25	H50	H75	H90
1	bone	83	52	24	3	100	97	41	8	100	96	44	6	100	100	17	0
2	breast	61	45	9	0	100	86	19	1	100	88	18	1	100	62	19	0
3	colorectal	79	53	28	2	100	98	47	4	99	96	49	4	100	95	32	3
4	esophagus	68	49	13	1	100	92	21	2	100	93	24	2	100	100	0	0
5	gastric	75	51	9	1	98	79	14	1	98	80	14	1	100	100	30	0
6	glioblastoma	94	85	41	2	100	98	43	3	100	98	48	2	NA	NA	NA	NA
7	glioma	85	72	34	2	100	99	45	4	100	100	47	3	100	100	38	5
8	kidney	63	45	12	3	100	92	12	8	100	96	27	6	NA	NA	NA	NA
9	leukemia	70	52	33	5	98	96	66	11	98	97	63	10	100	100	100	7
10	liver	60	49	12	1	100	92	25	2	100	92	23	2	NA	NA	NA	NA
11	lung	62	43	8	0	100	76	14	1	100	78	15	1	100	67	11	0
12	lung NSC	65	48	12	1	100	84	20	1	100	86	21	1	100	57	10	0
13	lung small	66	44	11	0	100	83	21	1	100	82	20	1	100	100	0	0
14	medulloblastoma	73	40	13	7	73	40	13	7	73	40	13	7	NA	NA	NA	NA
15	melanoma	64	46	15	3	100	85	29	5	100	87	28	5	NA	NA	NA	NA
16	ovary	61	42	6	0	100	76	8	1	99	78	11	1	100	80	7	0
17	pancreas	68	49	11	1	100	85	16	1	100	87	19	1	100	100	0	0
18	prostate	50	50	17	17	100	100	0	0	100	100	33	33	NA	NA	NA	NA
19	skin	67	48	12	2	100	87	20	4	100	89	22	4	100	100	100	0
20	thyroid	82	63	13	1	100	95	20	2	100	96	20	1	100	100	33	0
21	TNBC	62	45	8	1	100	84	17	0	99	86	16	1	100	50	17	0
22	urinary tract	66	54	10	2	97	85	13	2	95	84	15	2	100	67	0	0

Table 1: This table summarizes the CARNIVAL results for all 22 different types of cancer tested in CCLE. We report on the similarity of the optimized networks across four different settings and similarity cut-offs. The column names indicate first with a letter the type of filtering done (G=General,no filtering, M=Mutation Type, D= Deleterious function of mutation, H: Hotspot *TP53* mutation) and then with a number the similarity percentages (25,50,75 and 90%), or cut-offs. For example G25 reports the percentage of the networks when applying no filtering on which networks participate, that achieved at least 25% similarity score (see Methods). It is important to clarify that the first four columns include the number of all possible combinations of pairs of networks and thus the total number of comparisons induced is maximized, whereas in all other columns we move to a *conditional universe* since we filter for a specific *TP53* status each time; thus the number of total comparisons shrinks compared to the prior (general) pool. However, the percentage comparisons eventually phrase the results proportionally to the respective pool of total comparisons in each case.

	TCGA SUBTYPE	G25	G50	G75	G90	M25	M50	M75	M90	D25	D50	D75	D90	H25	H50	H75	H90
1	ACC	87	62	26	2	84	79	32	0	100	100	43	4	NA	NA	NA	NA
2	BLCA	83	50	19	1	94	75	24	2	100	93	35	3	97	75	19	1
3	BRCA_Basal	73	49	21	1	85	65	21	1	100	96	40	2	82	55	22	2
4	BRCA_Her2	72	47	17	1	87	72	17	1	100	93	33	2	75	43	14	0
5	BRCA_LumA	72	50	25	3	85	69	29	3	99	97	48	5	89	50	36	0
6	BRCA_LumB	68	52	23	2	87	80	34	3	100	98	43	3	64	44	13	2
7	BRCA_Normal	73	54	26	1	70	48	17	0	100	100	48	2	100	100	53	0
8	CESC_AdenoCarcinoma	79	57	21	0	100	100	40	0	100	100	38	0	NA	NA	NA	NA
9	CESC_SquamousCarcinoma	74	52	14	3	95	82	23	4	98	87	22	4	NA	NA	NA	NA
10	CHOL	67	40	0	0	100	83	0	0	100	86	0	0	NA	NA	NA	NA
11	COAD_CIN	82	50	33	4	87	62	38	4	100	97	65	7	81	50	31	3
12	COAD_GS	79	43	18	0	73	47	13	0	100	100	42	0	67	33	17	0
13	COAD_MSI	83	47	36	5	94	70	42	2	100	99	75	11	81	48	19	0
14	COAD_POLE	33	33	33	33	100	100	100	100	100	100	100	100	NA	NA	NA	NA
15	DLBC	100	40	40	0	100	100	100	0	100	100	100	0	100	100	100	0
16	ESCA_CIN	71	46	20	2	81	59	18	2	100	92	41	4	69	40	11	1
17	ESCA_ESCC	80	48	27	4	90	66	26	3	100	94	53	8	82	47	29	9
18	ESCA_POLE	100	67	0	0	100	67	0	0	100	67	0	0	NA	NA	NA	NA
19	GBM	100	100	20	0	100	100	17	0	100	100	20	0	100	100	0	0
20	GBM_IDHmut-non-codel	61	36	11	0	67	44	22	0	100	77	23	0	33	0	0	0
21	GBM_IDHwt	87	53	18	2	92	68	24	3	100	96	33	4	82	58	14	2
22	HNSC_HPV-	91	48	36	12	96	73	45	11	100	93	69	23	95	50	32	10
23	HNSC_HPV+	92	61	22	3	96	75	25	4	100	100	36	5	100	100	17	0
24	KICH	78	50	21	4	91	76	26	6	100	100	42	8	100	100	100	0
25	KIRC	81	45	15	4	100	90	23	3	100	92	32	8	NA	NA	NA	NA
26	KIRP	73	60	0	0	100	90	0	0	100	90	0	0	NA	NA	NA	NA
27	LAML	66	47	15	2	84	79	21	3	100	100	32	4	100	100	100	0
28	LGG_IDHmut-codel	52	52	24	0	67	67	27	0	100	100	45	0	50	50	17	0
29	LGG_IDHmut-non-codel	61	44	10	1	84	59	11	1	99	74	18	2	73	53	9	1
30	LGG_IDHwt	72	49	15	4	88	69	21	6	99	83	23	6	67	0	0	0
31	LIHC	76	45	14	1	91	73	19	1	99	87	27	2	67	38	5	0
32	LUAD	74	48	19	1	92	78	22	2	100	94	37	3	69	44	13	0
33	LUSC	84	48	26	4	95	80	33	4	100	92	49	7	77	50	17	1
34	MESO	71	46	11	1	96	72	13	0	100	91	22	2	67	33	0	0
35	OV	86	50	17	1	95	76	25	1	100	99	35	2	89	47	16	0
36	PAAD	97	49	34	8	99	78	44	9	100	97	66	15	96	51	29	6
37	PRAD	73	48	14	1	86	68	20	1	100	97	27	1	97	69	26	1
38	READ_CIN	81	49	30	4	85	62	34	5	100	98	58	7	78	49	28	5
39	READ_GS	100	0	0	0	NA	NA	NA	NA	NA	NA	NA	NA	NA	NA	NA	NA
40	READ_MSI	100	100	67	33	100	100	67	33	100	100	67	33	100	100	0	0
41	READ_POLE	33	33	33	33	100	100	100	100	100	100	100	100	NA	NA	NA	NA
42	SARC_DDLPS	100	0	0	0	NA	NA	NA	NA	NA	NA	NA	NA	NA	NA	NA	NA
43	SARC_LMS	82	46	18	1	86	60	16	1	100	94	37	3	64	43	11	0

Table 2 continued from previous page

	TCGA SUBTYPE	G25	G50	G75	G90	M25	M50	M75	M90	D25	D50	D75	D90	H25	H50	H75	H90
44	SKCM	75	48	13	0	87	71	18	1	100	97	27	1	0	0	0	0
45	STAD_CIN	73	47	18	1	83	63	17	1	100	93	37	2	72	43	14	0
46	STAD_EBV	100	100	100	0	NA	NA	NA	NA	100	100	100	0	NA	NA	NA	NA
47	STAD_GS	52	43	10	0	67	67	22	0	100	100	22	0	NA	NA	NA	NA
48	STAD_MSI	70	47	17	2	76	55	16	2	97	92	32	5	79	50	7	0
49	STAD_POLE	33	17	0	0	33	17	0	0	100	50	0	0	100	100	0	0
50	THYM	83	50	17	0	100	100	33	0	100	100	33	0	NA	NA	NA	NA
51	UCEC_CN_HIGH	84	55	18	1	90	75	26	1	99	98	32	2	78	52	14	0
52	UCEC_CN_LOW	89	50	14	0	100	100	25	0	100	100	28	0	NA	NA	NA	NA
53	UCEC_MSI	77	49	21	5	94	82	39	10	100	100	43	11	100	67	67	0
54	UCEC_POLE	82	49	20	3	87	66	29	4	100	100	41	5	100	100	33	33

Table 2: The CARNIVAL results on 54 different sub-types of cancer in TCGA, across different scores of similarity and different metrics such as mutation type or deleterious function of the gene. We report on the similarity of the optimized networks across four different settings and similarity cut-offs. The column names indicate first with a letter the type of filtering done (G=General,no filtering, M=Mutation Type, D= Deleterious function of mutation, H: Hotspot *TP53* mutation) and then with a number the similarity percentages (25,50,75 and 90%), or cut-offs. For example G25 reports the percentage of the networks when applying no filtering on which networks participate, that achieved at least 25% similarity score (see Methods). It is important to clarify that the first four columns include the number of all possible combinations of pairs of networks and thus the total number of comparisons induced is maximized, whereas in all other columns we move to a *conditional universe* since we filter for a specific *TP53* status each time; thus the number of total comparisons shrinks compared to the prior (general) pool. However, the percentage comparisons eventually phrase the results proportionally to the respective pool of total comparisons in each case.

1. XGBoost Classifier for mutation types

```
=============================
CCLE MUTATION TYPE CLASSIFIER
=============================

##### xgb.Booster
raw: 1000.1 Kb
call:
  xgb.train(params = params, data = dtrain, nrounds = nrounds,
    watchlist = watchlist, verbose = verbose, print_every_n = print_every_n,
    early_stopping_rounds = early_stopping_rounds, maximize = maximize,
    save_period = save_period, save_name = save_name, xgb_model = xgb_model,
    callbacks = callbacks, booster = "gbtree", nthread = 8, max_depth = 15,
    num_class = 7, objective = "multi:softmax")
params (as set within xgb.train):
  booster = "gbtree", nthread = "8", max_depth = "15", num_class = "7", objective = "multi:softmax", validate_
xgb.attributes:
  niter
callbacks:
  cb.print.evaluation(period = print_every_n)
  cb.evaluation.log()
# of features: 579
niter: 100
nfeatures : 579
evaluation_log:
    iter train_mlogloss
       1     1.33340279
       2     1.01552629
---
      99     0.09363334
     100     0.09358787
     Feature         Gain        Cover    Frequency
  1:   ZMAT3 1.111162e-02 1.339584e-02 0.0058375635
  2:   FOXK1 9.844554e-03 5.257179e-03 0.0031725888
  3:    CHN2 9.813659e-03 2.748695e-03 0.0012690355
  4:   DHRS2 9.468327e-03 1.132460e-02 0.0065989848
  5:  PLAGL1 9.101428e-03 9.337901e-03 0.0048223350
 ---
569:  DOCK10 5.059844e-06 1.295466e-04 0.0003807107
570:   ANXA2 2.590500e-06 7.435580e-05 0.0002538071
571:   DHRS3 2.267346e-06 2.239569e-04 0.0002538071
572:  BCL2L1 1.651103e-06 3.206996e-04 0.0002538071
573:    LRP5 1.438120e-06 1.972185e-05 0.0001269036

[1] "test-error= 0.384057971014493"

=============================
TCGA MUTATION TYPE CLASSIFIER
=============================

##### xgb.Booster
raw: 2.4 Mb
call:
  xgb.train(params = params, data = dtrain, nrounds = nrounds,
    watchlist = watchlist, verbose = verbose, print_every_n = print_every_n,
    early_stopping_rounds = early_stopping_rounds, maximize = maximize,
    save_period = save_period, save_name = save_name, xgb_model = xgb_model,
    callbacks = callbacks, booster = "gbtree", nthread = 8, max_depth = 15,
    num_class = 10, objective = "multi:softmax")
```

```
params (as set within xgb.train):
  booster = "gbtree", nthread = "8", max_depth = "15", num_class = "10", objective = "multi:softmax", validate
xgb.attributes:
  niter
callbacks:
  cb.print.evaluation(period = print_every_n)
  cb.evaluation.log()
# of features: 551
niter: 100
nfeatures : 551
evaluation_log:
    iter train_mlogloss
       1     1.51939502
       2     1.14208012
---
      99     0.05071035
     100     0.05065993
     Feature         Gain        Cover    Frequency
  1:     KEL 0.0047188423 0.0019719530 0.0019489453
  2:   SYNJ2 0.0047033423 0.0013614735 0.0014139407
  3:  EFCAB5 0.0045270220 0.0012962292 0.0024839499
  4:  TCEAL1 0.0043753315 0.0028048219 0.0023693060
  5:  CYP4F3 0.0043310066 0.0036889772 0.0030189544
 ---
546:  PDLIM1 0.0003518065 0.0006600330 0.0010700092
547:    XPO1 0.0003337655 0.0006941452 0.0014139407
548:  RECQL4 0.0002268286 0.0005200222 0.0010317946
549:   MSGN1 0.0002163415 0.0000634281 0.0003057169
550:     GML 0.0001988607 0.0001549554 0.0001910731

[1] "test-error= 0.384006334125099"
```

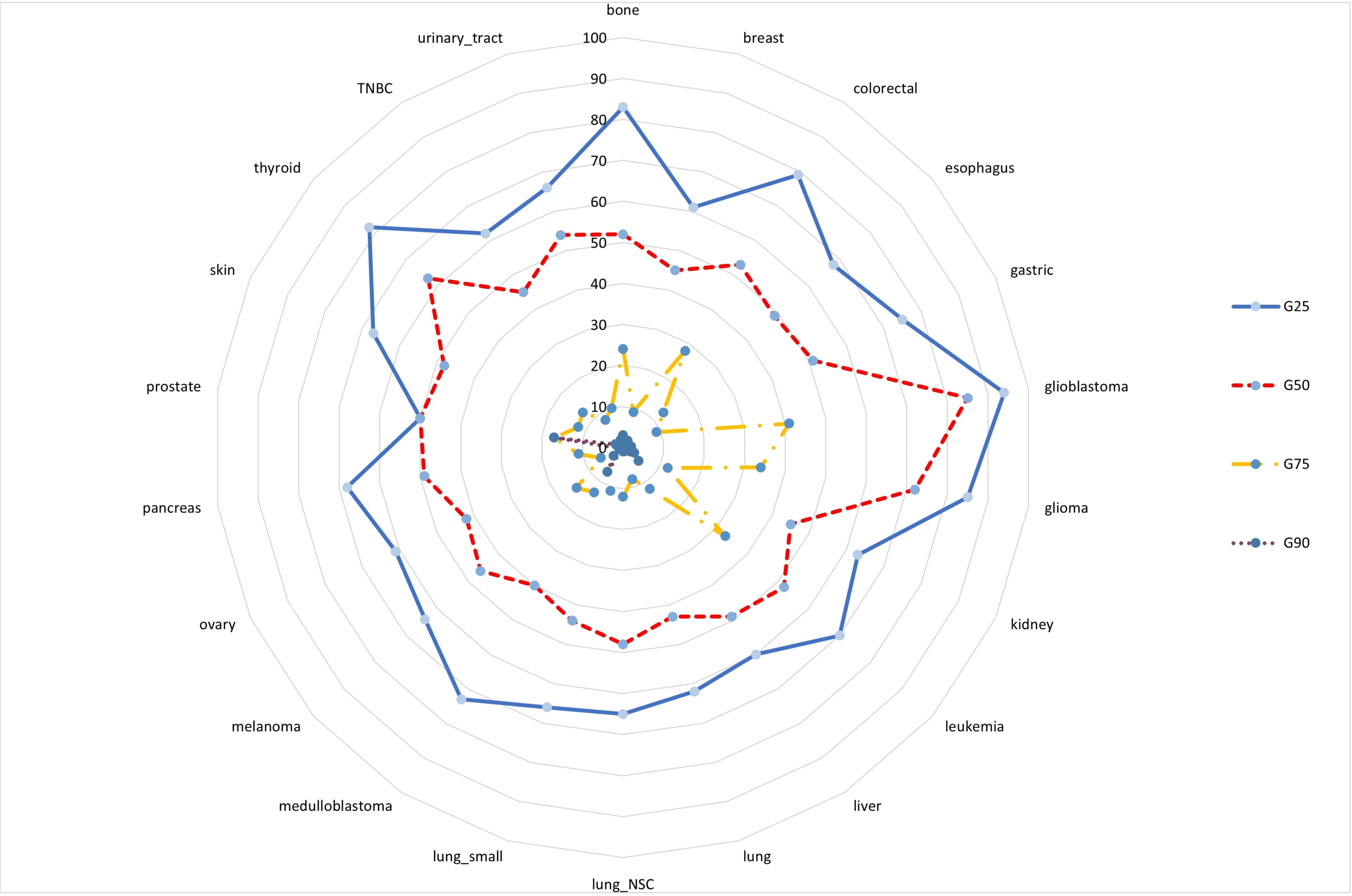

Supplemental Figure 13: CCLE : all networks compared

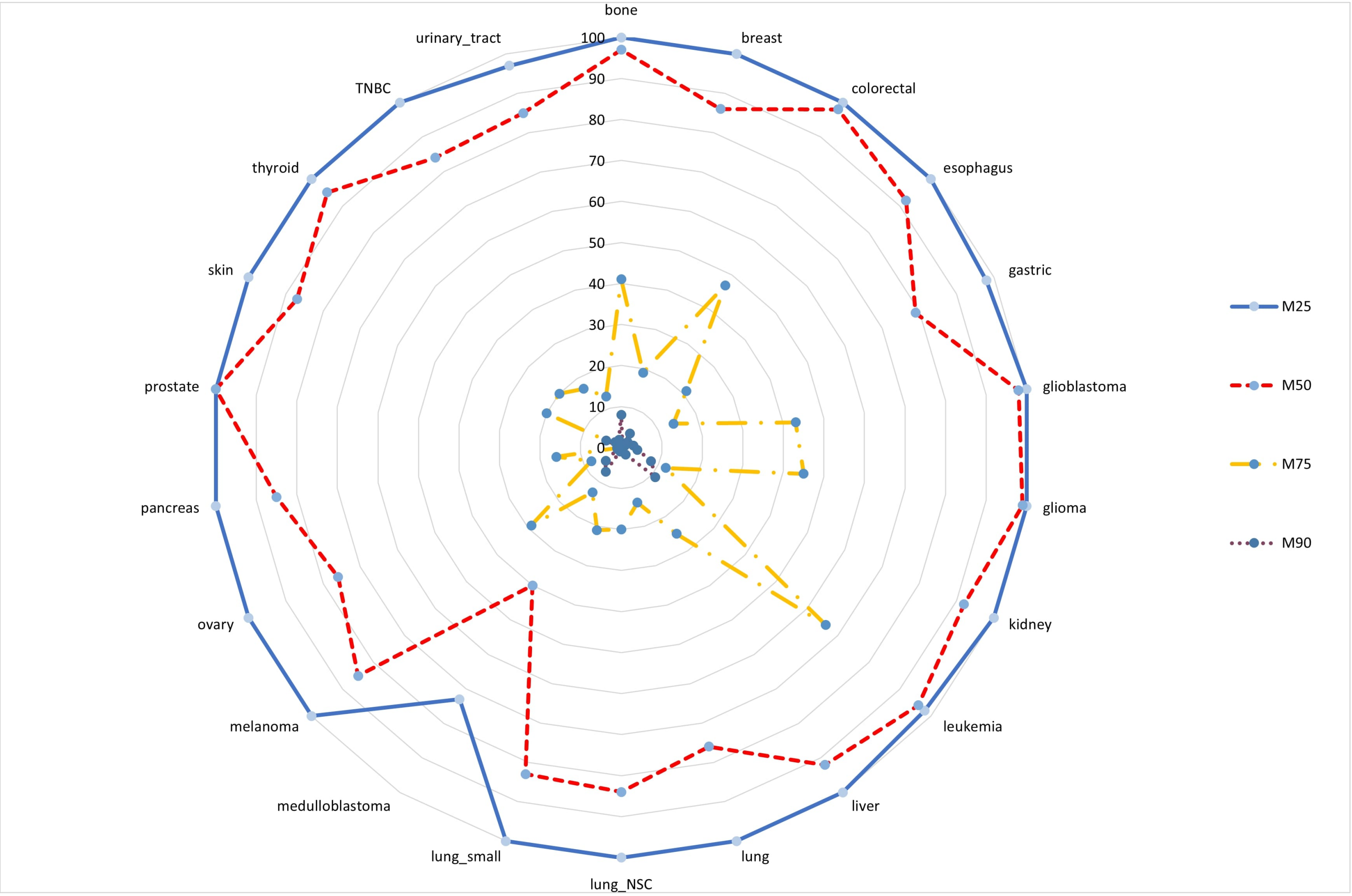

Supplemental Figure 14: CCLE : same mutation type

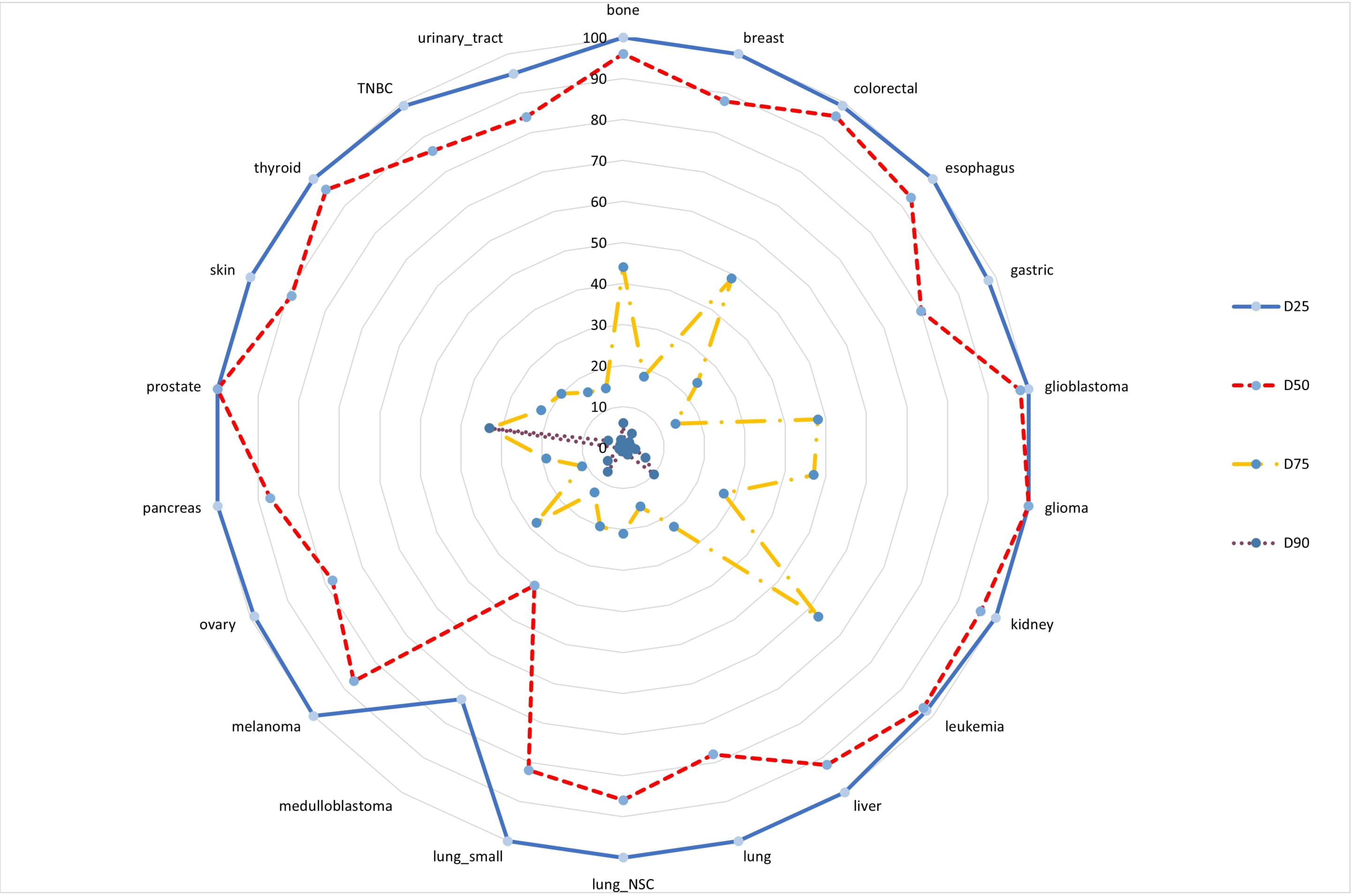

Supplemental Figure 15: CCLE : same deleterious function of mutation

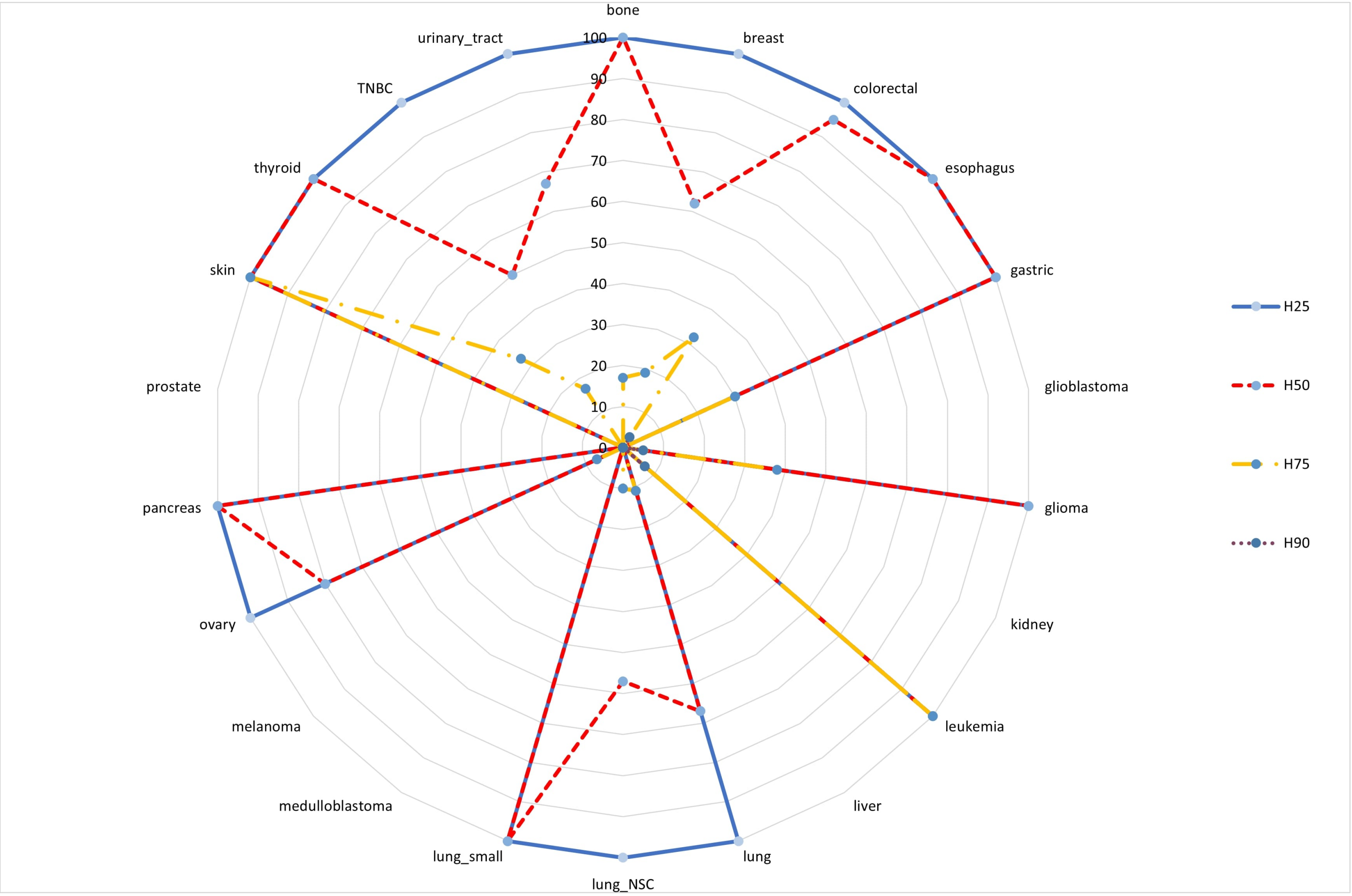

Supplemental Figure 16: CCLE : *TP53* hotspot mutation

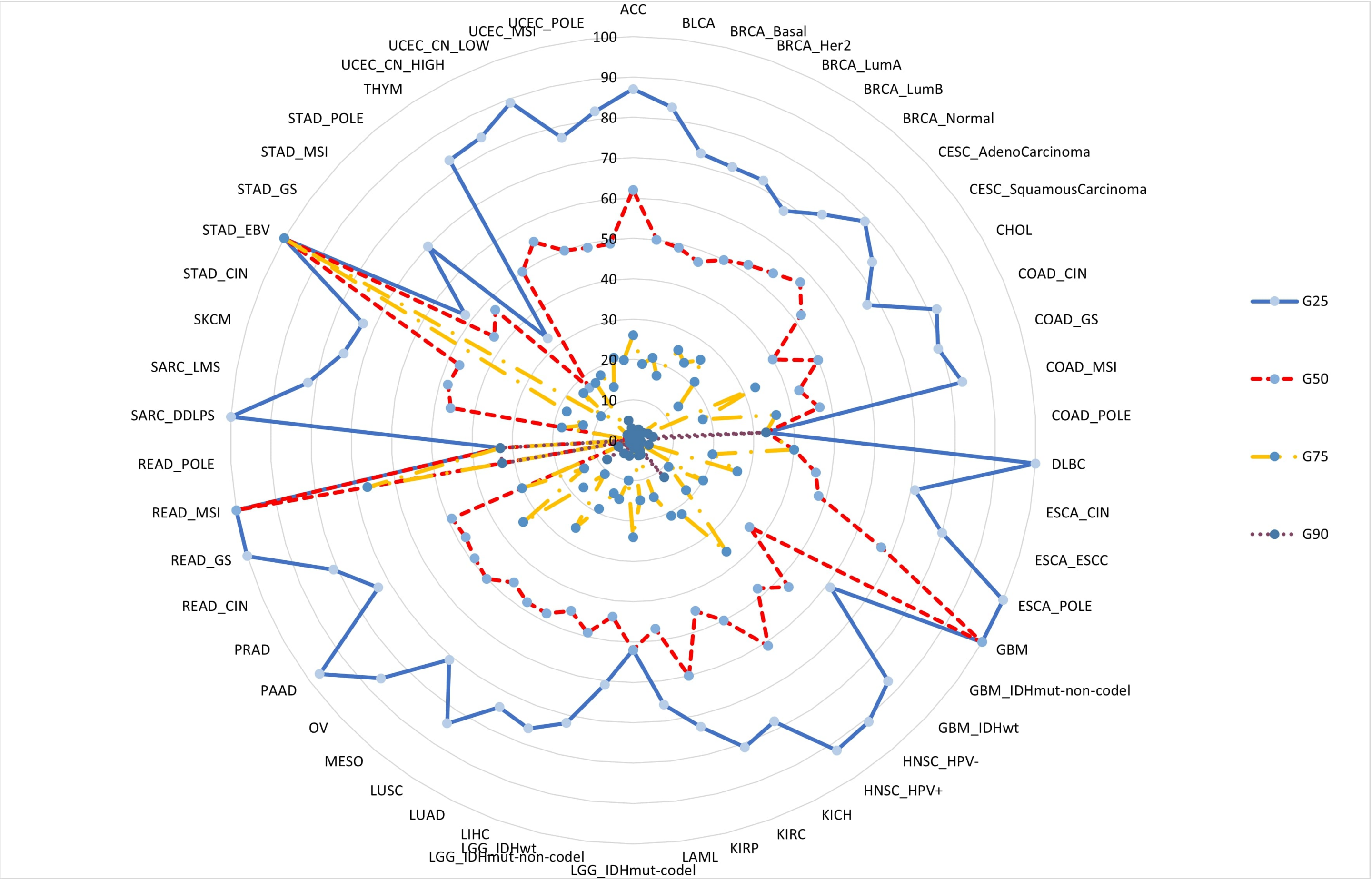

Supplemental Figure 17: TCGA : all networks compared

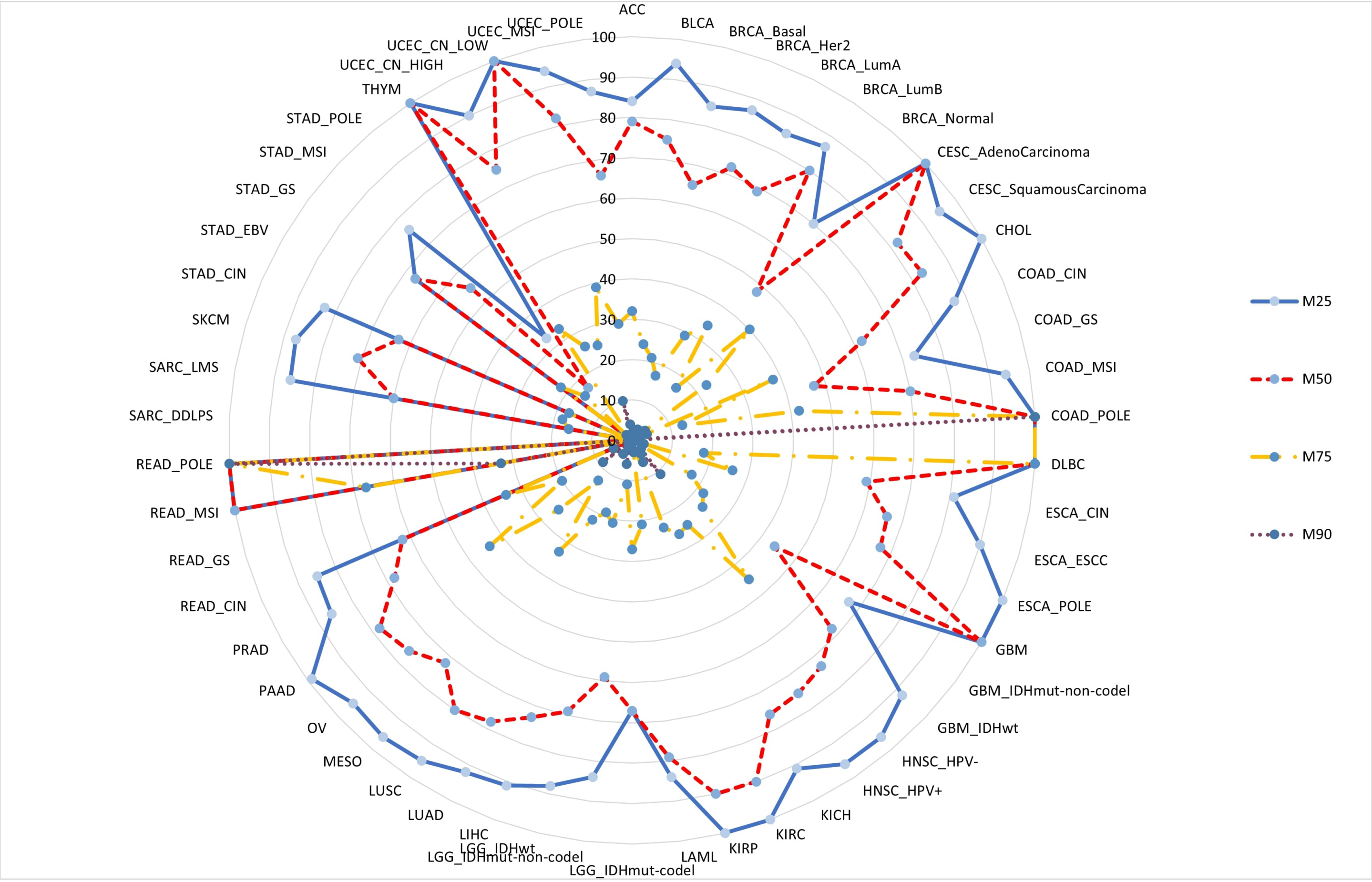

Supplemental Figure 18: TCGA : same mutation type

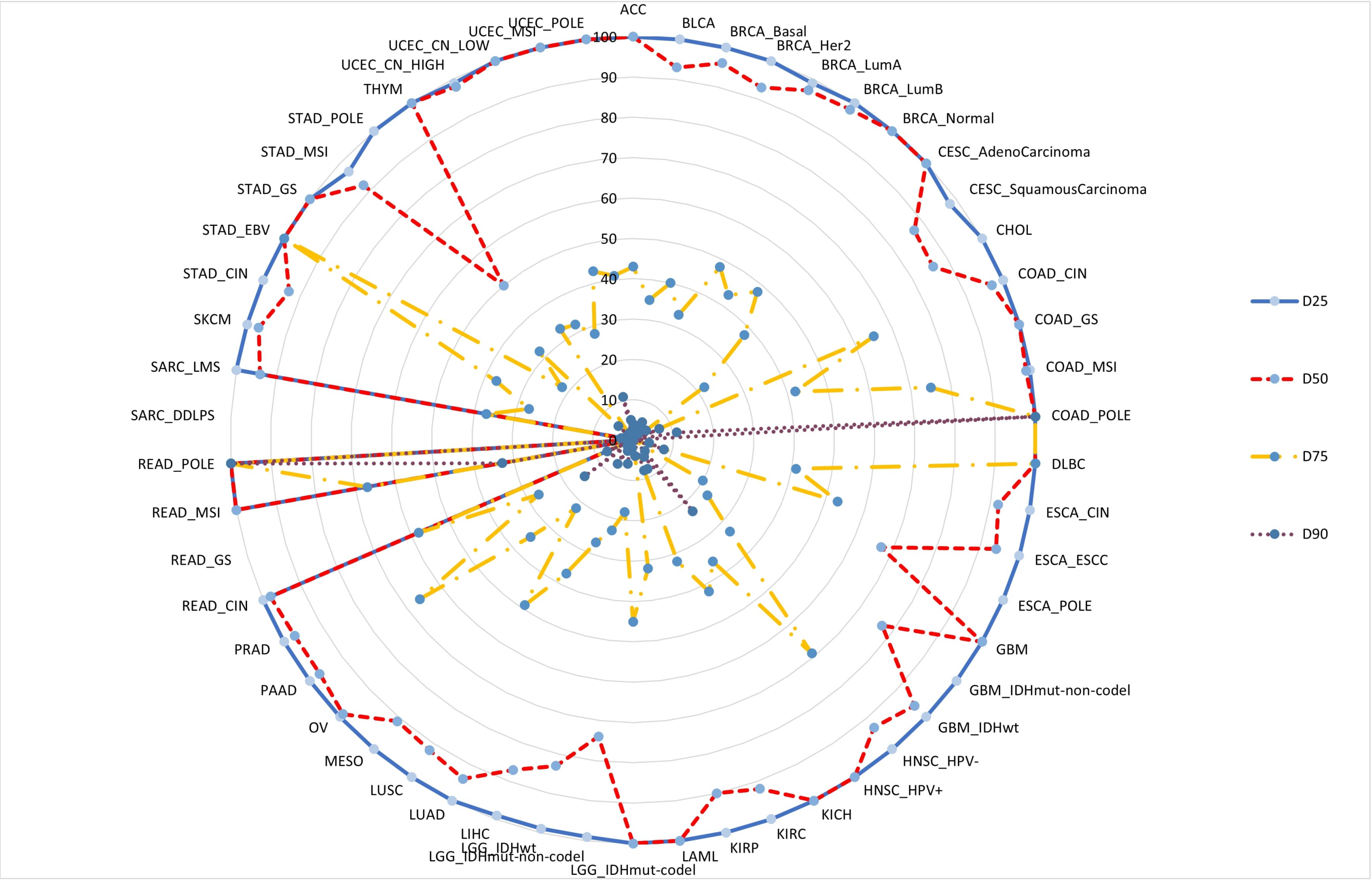

Supplemental Figure 19: TCGA : same deleterious function of mutation

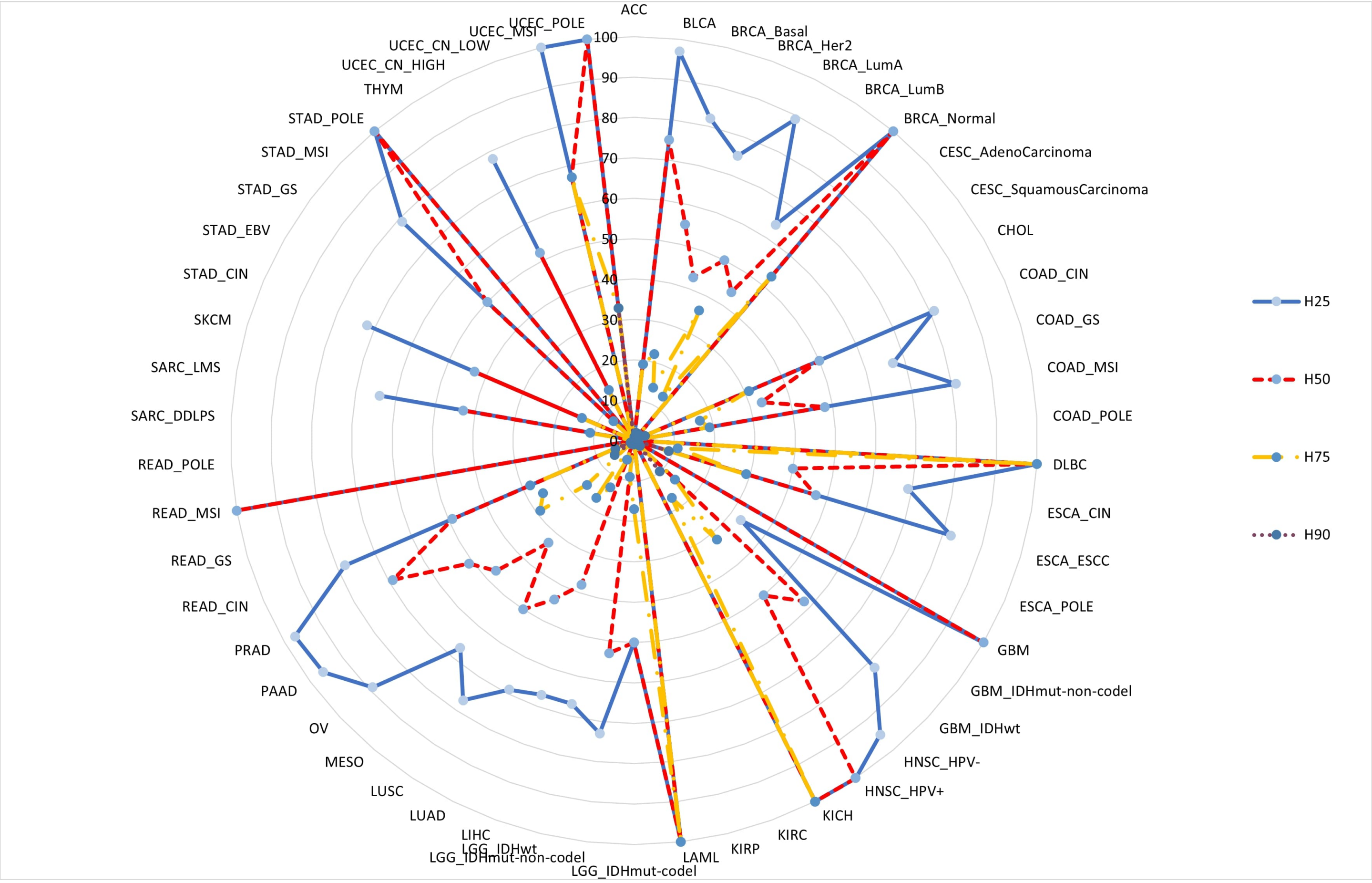

Supplemental Figure 20: TCGA : *TP53* hotspot mutation

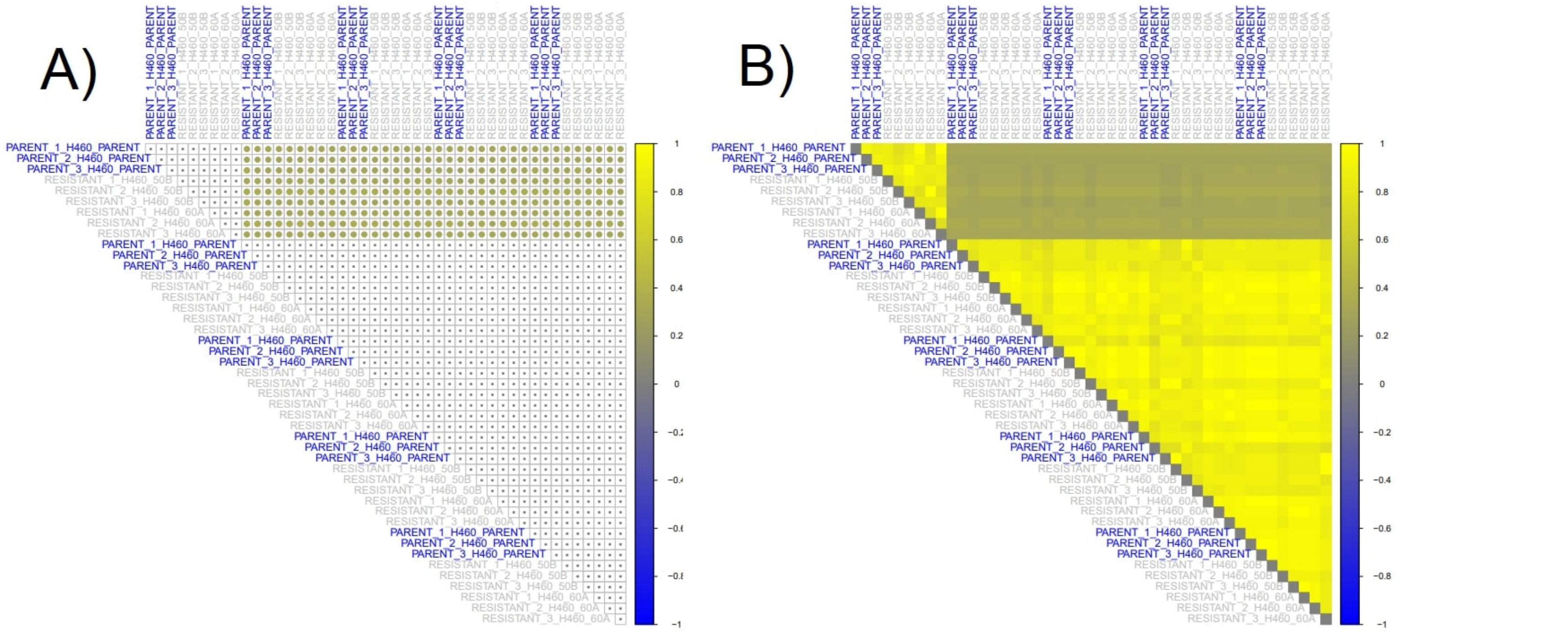

Supplemental Figure 21: Radiation experiment and the correlation matrix for the similarity of networks. A) only when comparing irradiated samples versus non-irradiated samples. The plot indicates that the compared networks exhibit very low similarity. More specifically for similarity cutoffs of 25,50,75 and 90%, the percentage of networks achieving at least this similarity are correspondingly 100%,0%,0%,0%. Only the first 9 rows were activated, as those are the ones accounting for samples with zero irradiation. B) When we compare all networks in pairs, without any filtering, the similarity for the same cutoffs are 100%, 67%, 67% and 36%. This indicates that the irradiated versus the samples under no exposure to radiation follow a different regulatory profile for the transcription factor *TP53* in our analysis.

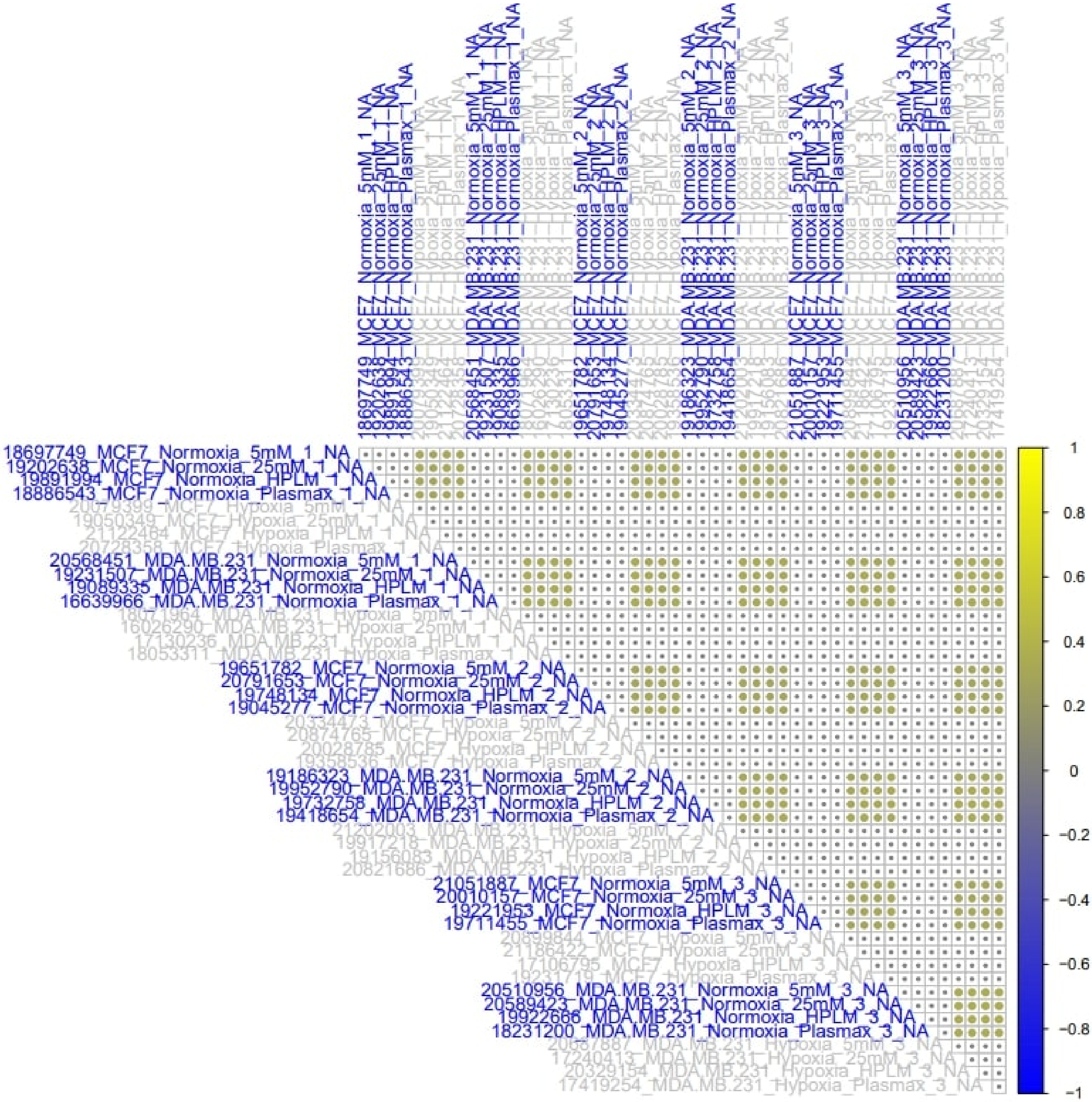

Supplemental Figure 22: In the small hypoxia experiment, the correlation plot shows the similarity of the networks only when comparing across hypoxic versus normoxic samples. We can see that the similarity diminished practically again to zero for similarity above 50% cutoff. For clarity, the normoxic samples are in blue color and the hypoxic in grey.

POST HOC TUKEY TEST FOR SUPPL.FIGURE 4a

```
Tukey multiple comparisons of means
    95% family-wise confidence level

$Variant_Classification
                                         diff         lwr        upr     p adj
Frame_Shift_Ins-Frame_Shift_Del      0.62005249 -0.44747760  1.6875826 0.6049127
In_Frame_Del-Frame_Shift_Del         2.29186046  0.86575237  3.7179685 0.0000493
Missense_Mutation-Frame_Shift_Del    2.31068749  1.78141115  2.8399638 0.0000000
Nonsense_Mutation-Frame_Shift_Del    0.32918173 -0.33266859  0.9910320 0.7625912
Splice_Site-Frame_Shift_Del          1.30335530  0.62417244  1.9825382 0.0000004
WT-Frame_Shift_Del                   1.65254668  1.11000055  2.1950928 0.0000000
In_Frame_Del-Frame_Shift_Ins         1.67180797  0.03397668  3.3096393 0.0418759
Missense_Mutation-Frame_Shift_Ins    1.69063499  0.72687128  2.6543987 0.0000057
Nonsense_Mutation-Frame_Shift_Ins   -0.29087076 -1.33334517  0.7516036 0.9824052
Splice_Site-Frame_Shift_Ins          0.68330281 -0.37026086  1.7368665 0.4695117
WT-Frame_Shift_Ins                   1.03249418  0.06137969  2.0036087 0.0287144
Missense_Mutation-In_Frame_Del       0.01882702 -1.33135973  1.3690138 1.0000000
Nonsense_Mutation-In_Frame_Del      -1.96267873 -3.37012915 -0.5552283 0.0008167
Splice_Site-In_Frame_Del            -0.98850516 -2.40418880  0.4271785 0.3753819
WT-In_Frame_Del                     -0.63931378 -1.99475730  0.7161297 0.8050570
Nonsense_Mutation-Missense_Mutation -1.98150575 -2.45822982 -1.5047817 0.0000000
Splice_Site-Missense_Mutation       -1.00733218 -1.50784127 -0.5068231 0.0000001
WT-Missense_Mutation                -0.65814081 -0.94723645 -0.3690452 0.0000000
Splice_Site-Nonsense_Mutation        0.97417357  0.33509474  1.6132524 0.0001539
WT-Nonsense_Mutation                 1.32336495  0.83194995  1.8147799 0.0000000
WT-Splice_Site                       0.34919137 -0.16532997  0.8637127 0.4115505
```

POST HOC TUKEY TEST FOR SUPPL.FIGURE 4b

Tukey multiple comparisons of means
95% family-wise confidence level

	diff	lwr	upr	p adj
Frame_Shift_Ins-Frame_Shift_Del	-0.179783068	-0.48276461	0.12319847	0.7107550
Fusion-Frame_Shift_Del	-0.404770490	-1.00600271	0.19646174	0.5284086
In_Frame_Del-Frame_Shift_Del	1.714206748	1.34899278	2.07942071	0.0000000
In_Frame_Ins-Frame_Shift_Del	0.942046895	0.14923290	1.73486089	0.0061778
Missense_Mutation-Frame_Shift_Del	1.683158612	1.52601736	1.84029986	0.0000000
Nonsense_Mutation-Frame_Shift_Del	-0.007396685	-0.20001891	0.18522554	1.0000000
Splice_Region-Frame_Shift_Del	0.144974812	-0.29265986	0.58260949	0.9929885
Splice_Site-Frame_Shift_Del	0.452805389	0.22447302	0.68113776	0.0000000
Translation_Start_Site-Frame_Shift_Del	1.428113657	-1.49068267	4.34690998	0.8929715
WT-Frame_Shift_Del	1.294989158	1.14460220	1.44537611	0.0000000
Fusion-Frame_Shift_Ins	-0.224987422	-0.86541067	0.41543583	0.9889118
In_Frame_Del-Frame_Shift_Ins	1.893989816	1.46732485	2.32065478	0.0000000
In_Frame_Ins-Frame_Shift_Ins	1.121829962	0.29889875	1.94476118	0.0005915
Missense_Mutation-Frame_Shift_Ins	1.862941680	1.59210016	2.13378319	0.0000000
Nonsense_Mutation-Frame_Shift_Ins	0.172386382	-0.12047037	0.46524313	0.7208669
Splice_Region-Frame_Shift_Ins	0.324757879	-0.16532973	0.81484549	0.5531471
Splice_Site-Frame_Shift_Ins	0.632588456	0.31510235	0.95007456	0.0000000
Translation_Start_Site-Frame_Shift_Ins	1.607896725	-1.31922366	4.53501711	0.7989456
WT-Frame_Shift_Ins	1.474772225	1.20779284	1.74175161	0.0000000
In_Frame_Del-Fusion	2.118977238	1.44687206	2.79108241	0.0000000
In_Frame_Ins-Fusion	1.346817384	0.37373046	2.31990430	0.0004367
Missense_Mutation-Fusion	2.087929102	1.50223507	2.67362313	0.0000000
Nonsense_Mutation-Fusion	0.397373804	-0.19882033	0.99356794	0.5441067
Splice_Region-Fusion	0.549745301	-0.16430528	1.26379589	0.3172243
Splice_Site-Fusion	0.857575878	0.24890536	1.46624640	0.0003021
Translation_Start_Site-Fusion	1.832884147	-1.13994550	4.80571379	0.6597150
WT-Fusion	1.699759647	1.11584153	2.28367776	0.0000000
In_Frame_Ins-In_Frame_Del	-0.772159854	-1.61998012	0.07566041	0.1131148
Missense_Mutation-In_Frame_Del	-0.031048136	-0.37007402	0.30797775	1.0000000
Nonsense_Mutation-In_Frame_Del	-1.721603434	-2.07846265	-1.36474422	0.0000000
Splice_Region-In_Frame_Del	-1.569231937	-2.10005139	-1.03841249	0.0000000
Splice_Site-In_Frame_Del	-1.261401360	-1.63873523	-0.88406749	0.0000000
Translation_Start_Site-In_Frame_Del	-0.286093091	-3.22030800	2.64812182	0.9999999
WT-In_Frame_Del	-0.419217591	-0.75516612	-0.08326906	0.0028942
Missense_Mutation-In_Frame_Ins	0.741111717	-0.03998452	1.52220796	0.0810455
Nonsense_Mutation-In_Frame_Ins	-0.949443580	-1.73844376	-0.16044340	0.0051186
Splice_Region-In_Frame_Ins	-0.797072083	-1.67851526	0.08437109	0.1194534
Splice_Site-In_Frame_Ins	-0.489241506	-1.28771106	0.30922805	0.6682994
Translation_Start_Site-In_Frame_Ins	0.486066763	-2.53134768	3.50348121	0.9999883
WT-In_Frame_Ins	0.352942263	-0.42682322	1.13270774	0.9335407
Nonsense_Mutation-Missense_Mutation	-1.690555298	-1.82715922	-1.55395137	0.0000000
Splice_Region-Missense_Mutation	-1.538183800	-1.95421435	-1.12215325	0.0000000
Splice_Site-Missense_Mutation	-1.230353223	-1.41390970	-1.04679674	0.0000000
Translation_Start_Site-Missense_Mutation	-0.255044955	-3.17068027	2.66059036	1.0000000
WT-Missense_Mutation	-0.388169454	-0.45277245	-0.32356646	0.0000000
Splice_Region-Nonsense_Mutation	0.152371497	-0.27831559	0.58305859	0.9882982
Splice_Site-Nonsense_Mutation	0.460202074	0.24548614	0.67491800	0.0000000
Translation_Start_Site-Nonsense_Mutation	1.435510343	-1.48225237	4.35327305	0.8894635
WT-Nonsense_Mutation	1.302385843	1.17360892	1.43116277	0.0000000
Splice_Site-Splice_Region	0.307830577	-0.13996817	0.75562933	0.4953931
Translation_Start_Site-Splice_Region	1.283138846	-1.66096713	4.22724483	0.9479886
WT-Splice_Region	1.150014346	0.73648770	1.56354099	0.0000000
Translation_Start_Site-Splice_Site	0.975308269	-1.94502931	3.89564585	0.9925236
WT-Splice_Site	0.842183769	0.66437532	1.01999222	0.0000000

```
WT-Translation_Start_Site                -0.133124500 -3.04840359  2.78215459 1.0000000
```

Pairwise comparisons between group levels with corrections for multiple testing for Suppl.Figure 9 using Bonferroni p-value adjustment

```
Pairwise comparisons using Log-Rank test
  1       2       3
2 < 2e-16 -       -
3 0.92    < 2e-16 -
4 0.92    2.4e-13 0.94

P value adjustment method: BH
```
